# Supplementary material for: Treatment of Macular Degeneration Using Embryonic Stem Cell-Derived Retinal Pigment Epithelium: Preliminary Results in Asian Patients
Source: Stem Cell Reports. 2015 Apr 30;4(5):860–72. doi: 10.1016/j.stemcr.2015.04.005 (PMC4437471; doi:10.1016/j.stemcr.2015.04.005)
Supplement: Document S1. Supplemental Experimental Procedures, Figure S1, and Tables S1 and S2 [file mmc1.pdf]

Stem Cell Reports, Volume *4*

Supplemental Information

Treatment of Macular Degeneration Using Embryonic Stem Cell-Derived Retinal Pigment Epithelium: Preliminary Results in Asian Patients

Won Kyung Song, Kyung-Mi Park, Hyun-Ju Kim, Jae Ho Lee, Jinjung Choi, So Young Chong, Sung Han Shim, Lucian V. Del Priore, and Robert Lanza

Figure S1.

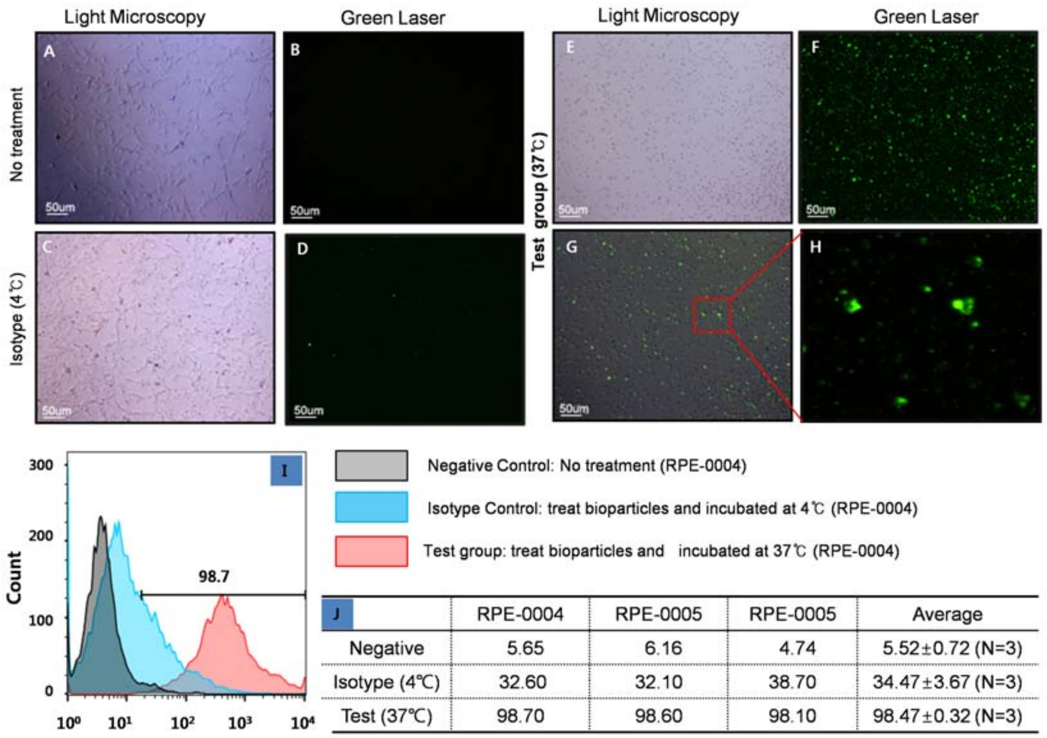

	RPE-0004	RPE-0005	RPE-0005	Average
Negative	5.65	6.16	4.74	5.52±0.72 (N=3)
Isotype (4℃)	32.60	32.10	38.70	34.47±3.67 (N=3)
Test (37℃)	98.70	98.60	98.10	98.47±0.32 (N=3)

Figure S1. Quantification of Phagocytosis Assay Using FACS Analysis, Related to Results section. Frozen vials of final product were thawed and divided into three experiment groups: No treatment (A, B); treated with fluorescent *E.coli* bioparticles and incubated at 4℃ (C, D); and treated with fluorescent *E.coli* bioparticles and incubated at 37℃ (E-H). Phagocytized resuts of the hRPE cells were shown in the image of light microscopy (E), green laser (F) and merged in (G). Insert reveal magnified area for internalization of bioparticles clearly shown in (H). FACS analysis results of lot# RPE-0004 shown in (I) as an representative and the quantification of phagocytosis assay for the 3 lots of hRPE products shown in table (J). Scale bars: 50um.

Table S1. Schedule of Assessments for AMD Study, Related to Experimental Procedures Section.

Study Day / Assessment	Screening	Baseline	Day of Transplant	Post-transplant Assessments													
	-30 to -1D	-7 to -1D	D 0	Day 1	Day 3	Day 7±1D	Week 2±3D	Week 3±3D	Week 4±3D	Week 6±5D	Week 8±5D	Week 13±5D	Week 15±5D	Week 17±5D	Week 26±7D	Week 39±7D	Week 52±7D
Sign informed consent	X																
Assess inclusion/exclusion	X	X															
Blood collection for xenogeneic transplantation archiving		X															
Medical history	X	X	X	X	X	X	X	X	X	X	X	X	X	X	X	X	X
Review of adverse events		X	X	X	X	X	X	X	X	X	X	X	X	X	X	X	X
Concomitant medications	X	X	X	X	X	X	X	X	X	X	X	X	X	X	X	X	X
Physical examination, vital signs	X			X	X	X	X	X	X	X	X	X	X	X	X	X	X
Serum pregnancy test	X	X															X
Clinical laboratory tests	X			X	X	X	X	X	X	X	X	X	X	X	X	X	X
Cancer screening	X																
Chest x-ray	X																
ECG	X			X							X	X			X	X	X
Visual acuity	X	X		X	X	X	X	X	X	X	X	X	X	X	X	X	X
Refraction	X	X		X	X	X	X	X	X	X	X	X	X	X	X	X	X
Tonometry	X	X		X	X	X	X	X	X	X	X	X	X	X	X	X	X
NEI visual function questionnaire VFQ-25	X	X						X	X	X	X	X		X			X
Full dilated slit lamp evaluation	X	X		X	X	X	X	X	X	X	X	X	X	X	X	X	X
Indirect ophthalmoscope	X	X		X	X	X	X	X	X	X	X	X	X	X	X	X	X
Visual field testing	X	X					X	X	X	X	X	X		X	X	X	X
Spectral Domain OCT		X		X		X	X	X	X	X	X	X	X	X	X	X	X
Autofluorescence photography		X							X			X			X		X
Fundus photography		X		X				X	X	X	X	X	X	X	X	X	X
Fluorescein angiography		X						X	X		X	X		X	X	X	X
Electroretinogram		X										X			X		X
Reading speed		X							X			X			X		X
Axial Length Measurement	X																
Tacrolimus administration		X	X	X	X	X	X	X	X								
Tacrolimus blood		X	X	X	X	X	X	X	X								
Mycophenolate mofetil administration		X	X	X	X	X	X	X	X	X	X	(X)	(X)	(X)	(X)	(X)	(X)
Hospitalization		X	X	X													

Table S2. Schedule of Assessments for the SMD Study, Related to Experimental Procedures Section.

Study Day / Assessment	Screening	Baseline	Day of Transplant	Post-transplant Assessments											
	-30 to -1D	-7 to -1D	D 0	Day 1	Day 3	Day 7±1D	Week 2±3D	Week 3±3D	Week 4±3D	Week 6±5D	Week 8±5D	Week 13±5D	Week 26±7D	Week 39±7D	Week 52±7D
Sign informed consent	X														
Assess inclusion/exclusion	X	X													
Blood collection for xenogeneic transplantation archiving		X													
Medical history	X	X	X	X	X	X	X	X	X	X	X	X	X	X	X
Review of adverse events		X	X	X	X	X	X	X	X	X	X	X	X	X	X
Concomitant medications	X	X	X	X	X	X	X	X	X	X	X	X	X	X	X
Physical examination, vital signs	X			X	X	X	X	X	X	X	X	X	X	X	X
Serum pregnancy test	X	X													X
Clinical laboratory tests	X			X	X	X	X	X	X	X	X	X	X	X	X
Cancer screening	X														
Chest x-ray	X														
ECG	X			X							X	X		X	X
Visual acuity	X	X		X	X	X	X	X	X	X	X	X	X	X	X
Refraction	X	X		X	X	X	X	X	X	X	X	X	X	X	X
Tonometry	X	X		X	X	X	X	X	X	X	X	X	X	X	X
NEI visual function questionnaire VFQ-25	X	X						X	X	X	X	X			X
Full dilated slit lamp evaluation	X	X		X	X	X	X	X	X	X	X	X	X	X	X
Indirect ophthalmoscope	X	X		X	X	X	X	X	X	X	X	X	X	X	X
Visual field testing	X	X					X	X	X	X	X	X		X	X
Spectral domain OCT		X		X		X	X	X	X	X	X	X	X	X	X
Autofluorescence photography		X							X			X			X
Fundus photography		X		X				X	X	X	X	X	X	X	X
Fluorescein angiography		X						X	X		X	X		X	X
Electroretinogram		X										X			X
Tacrolimus administration		X	X	X	X	X	X	X	X						
Tacrolimus blood		X	X	X	X	X	X	X	X						
Mycophenolate mofetil administration		X	X	X	X	X	X	X	X	X	X	(X)	(X)	(X)	
Hospitalization		X	X	X											

Supplemental Experimental Procedures

Phagocytosis Assay, Related to Results section.

Fluorescent *E.coli* Bioparticles (Invistrogen Cat #V6694) suspension was prepared prior to

phagocytosis assay according to the manufacture's instruction. Differentiated hRPE cells during the culture in a 6-well plate were incubated with 600μl/well fluorescent labeled bioparticles overnight at 37℃. After washing cells twice with 2ml of phosphate-buffered saline (PBS pH7.4), 2ml of cell culture medium was added to each well and incubated at 37℃ for 2 days. For the final product, frozen vials were thawed, washed twice with culture medium and cells were divided into three 15ml Falcon tubes. 200ul cell culture medium was added for one tube and 200ul fluorescent *E.coli* bioparticles were added for the other two tubes. Except isotype negative control tube, the other tubes were incubated at 37℃ for overnight. As for an isotype negative control tube, which was treated with 200μl fluorescent labeled bioparticles, was incubated at 4℃. After removing the biopartlcles solutions, cells were washed twice with 10ml cell culture medium, seeded 5x10^5 cells/well of 6-well plate and incubated at 37℃ for 2 days. For fluorescence observation, the cells were washed twice with 2ml of phosphate-buffered saline (PBS pH 7.4) and washed once with 1ml purified water. The cells then were fixed with 400μl of 4% paraformaldehyde solution for 5-10 minutes. The cells were examined under the fluorescence microscopy (Nikon ECLIPSE Ti-U, S/N: 633088) after removing fixative solution and were washed twice with 100μl phosphate-buffered saline (PBS pH 7.4). Photo images were taken by using NIS-Elements BR (version:3.2.3) software. For quantification analysis of the phagocytosis, the rest of the cells in the 6-well plate were trypsinized with 0.25% Trypsin-EDTA and underwent FACS analysis using Guaca easyCyteTM Dual Laser System and Guva Software (version 2.2.3).

FACS analysis, Related to Results section.

For OCT-4 staining, 1 x 10^5 cells of each sample were washed with 10% FBS/DMEM:F12 and resuspended in 1ml 4% paraformaldehyde/PBS and incubated for 10min at room temperature. The cells were washed twice with 10% FBS/DMEM:F12 and resuspended in 0.1% Triton X-100/PBS and incubated for 15 minutes on ice. After washing twice with 10% FBS/DMEM:F12, the cells were resuspended in 95μl phosphate-buffered saline (PBS; pH 7.4) and incubated with 0.1 μg of the primary antibody (OCT-3/4-Alex 488, BD Biosciences cat# 560217) against OCT-3/4 for 30~60 minutes at 4°C in dark. For TRA-1-60 staining, the cells were washed twice with 1 ml PBS supplemented with 1% heat inactivated FBS. 1 x 10^5 cells of each sample were resuspended in 95 μl phosphate-buffered saline (PBS; pH 7.4) and incubated with 0.1 μg of the primary antibody (TRA-1-

60-PE, BD Biosciences cat #560193) against TRA-1-60 for 30~60 minutes at 4°C in dark. The negative controls used for FACS gating were Mouse IgG1K Isotype Control FITC (e-Bioscience cat#12-4714) and Mouse IgG1K Isotype Control PE (e-Bioscience cat#12-4714). Each sample was washed twice with 1 ml PBS supplemented with 1% heat inactivated FBS. Finally, the cells were resuspended in 400 µl PBS and subsequently analyzed by flow cytometry using Guaca easyCyteTM Dual Laser System and Guava soft software(version 2.2.3). The sample flow rate during analysis did not exceed 300-400 cells per second.

Immunocytochemistry, Related to Results section.

Cells were fixed with 4 % paraformaldehyde for 5-10 minutes and permeabilized with 0.2 % Triton x-10 in PBS for 10 minutes. Unspecific binding was blocked in PBS supplemented with 10 % goat serum for 1-2 hours, and incubated with primary antibodies for 1 hour at room temperature or overnight at 4°C in blocking buffer. After washing twice with phosphate-buffered saline (PBS; pH 7.4), the cells were incubated with fluorescent secondary antibodies Alexa Fluor® 488 conjugate or Alexa Fluor® 594 conjugate (Invitrogen cat# A11008, cat# A11005) for 1 hour at room temperature. Nuclei were stained with 4'-6-Diamidino-2-phenylindole (DAPI, Sigma cat# D9564) for 5 min at room temperature. The cells were then examined under the fluorescence microscopy (Nikon ECLPSE Ti-U, S/N:633088). Photo images were taken by using NIS-Elements BR (version:3.2.3) software. Primary antibodies for the staining as follows: ZO-1 (Invitrogen cat# 40-2300), PAX-6 (Millipore cat# AB2237), MITF (Thermo cat# MS-772-p), Bestrophin (Novus Biologicals cat# NB300-164), OCT-3/4 (SantaCruz cat# SC5279), NANOG (CellSignaling #3580S).

Real-time PCR, Related to Results section.

The RNeasy RNA isolation kit form Qiagen was used to extract total cellular RNA, and cNDA was synthesized from the RNA with the cDNA synthesis kit from Qiagen. Quantitative real-time PCR was performed using the Roche LihgtCycler 480/96 Quantitative Real-Time PCR Instrument and TaqMan® Gene Expression Maste Mix, following manufacturer's cycle conditions. *NANOG, OCT-4* and *SOX2* for hES markers and *RPE-65, PAX-6, MITF* and *BESTROPHIN* for hRPE markers were

analyzed. Primers for hES and hRPE markers were generated by Ocata therapeutics Inc.' as custom-made inventoried from Applied Biosystems (*NANOG*: cat# Hs02387400, *OCT-4*: cat# Hs03005111, *SOX-2*: cat# Hs01053049, *RPE-65*: cat#Hs01071462, *PAX-6*: cat#Hs00240871, *MITF*: cat#Hs01117294, *Bestrophin*: cat#Hs00188249). As an endogenous control, human *beta ACTIN* was used (Applied Biosystems: cat# 4333762T).

Safety analysis, Related to Results section.

For adventitious viruses test, we used the methods of adsorption of hemagglutininis virus by red blood cell. Endotoxin test was measured by EndoScan-V (version 4.0 and higher) with the Endosafe®-MSC kinetic Reader (Charles River Laboratories, Model no. MCS Reader, S/N: 0314). Releasing specification for final product is < 0.5 EU/ml and the result for clinical product is normally < 0.32 EU/ml. MAP test for murine viruses was conducted by Charles River Laboratories (251 Ballardvale Street Wilmington, MA01887 USA) for the three different lots.

Viability test after final formulation, Related to Results section.

Live cells were counted in a hemocytometer using 0.4% trypan blue dye for measure viability of cells. After final washing in BSS plus solution, the cells were suspended in about 200μl and count the cells to make 2,000 cells/μl. And then, the cells were manipulated to loading density 444 cells/μl targeting for the clinical does 50,000 cells/150μl. Final formulated cells were stored at 2-8□ until clinical use up to 4 hours.

Inclusion/Exclusion Criteria for AMD Study, Related to Experimental Procedures Section.

Inclusion Criteria:

1. Adult male or female older than 55 years of age.
2. Patient should be in sufficiently good health to reasonably expect survival for at least 4 years.
3. Clinical findings consistent with advanced dry age-related macular degeneration (AMD) with evidence of one or more areas of >250 microns of geographic atrophy (as defined in the Age-related Eye Disease Study [AREDS] study) involving the central fovea.

4. Geographic atrophy (GA) defined as attenuation or loss of RPE as observed by slit-lamp biomicroscopy, optical coherence tomography (OCT), and fluorescein angiography (FA).
5. The visual acuity (Best Corrected Visual Acuity [BCVA]) of the eye to receive the transplant will be no better than 20/400.
6. The visual acuity (BCVA) of the eye that is NOT to receive the transplant will be no worse than 20/400.
7. Electrophysiological findings consistent with advanced dry AMD.
8. Medically suitable to undergo vitrectomy and subretinal injection.
9. Medically suitable for general anesthesia.
10. If female and of childbearing potential, willing to use medically effective forms of birth control during the study.

    *Medically effective method of contraception: condoms, continuous use oral contraceptives for more than 3 months, injection or insertion of contraceptives, and insertion of an intrauterine device (IUD).
11. If male, willing to use medically effective contraception during the study.
12. Willing to defer all future blood, blood components or tissue donations.
13. Able to understand and willing to sign the informed consent form.

Exclusion Criteria:

1. Presence of active or inactive choroidal neovascularization(CNV).
2. Presence or history of retinal dystrophy, retinitis pigmentosa, chorioretinitis, central serous chorioretinopathy, diabetic retinopathy or other retinal vascular or degenerative disease other than AMD.
3. History of optic neuropathy
4. Presence of macular atrophy due to causes other than AMD.
5. Presence of glaucomatous optic neuropathy in the study eye, uncontrolled intraocular pressure (IOP), or the use of two or more agents to control IOP (acetazolamide, beta blocker, alpha-1-agonist, prostaglandins, carbonic anhydrase inhibitors).
6. Cataract of sufficient severity likely to necessitate surgical extraction within 1 year.
7. History of retinal detachment repair in the study eye.

8. Axial myopia of greater than -8 diopters.
9. Axial length greater than 28 mm.
10. Any other sight-threatening ocular disease.
11. Any history of retinal vascular disease (compromised blood-retinal barrier).
12. History of glaucoma.
13. Uveitis or other intraocular inflammatory disease.
14. Significant lens opacities or other media opacity.
15. Ocular lens removal within previous 3 months.
16. Ocular surgery in the study eye in the previous 3 months
17. History of malignancy.
18. Medically not suitable for transplantation of an embryonic stem cell line: Any laboratory value that falls slightly outside of the normal range will be reviewed by the medical monitor and investigators to determine its clinical significance.
    1) History of drug abuse
    2) Positive human immunodeficiency virus (HIV), hepatitis B (HBV), and hepatitis C (HCV) serologies.
    3) Any immunodeficiency.
    4) Positive cancer screening test
        - Medical history & physical examination
        - Chest X-ray
        - Complete blood count (CBC)
        - Routine urinalysis (U/A)
        - Thyroid exam (T3, T4, TSH, thyroid ultrasonography)
        - If male, testicular examination (scrotal ultrasonography, LDH, beta-HCG)
        - If male, digital rectal examination (DRE) and prostate specific antigen (PSA) testing
        - Stomach cancer examination (upper gastrointestinal series or esophagogastroduodenoscopy
        - Liver cancer examination (abdomen ultrasonography+ α-fetoprotein)

- Colorectal cancer examination (occult blood in feces + colonoscopy or colon study)
- Breast cancer examination (mammography, breast ultrasonography, breast physical examination)
- Uterine cervical cancer examination (sonohysterography, Pap Smear, pelvis examination)

5) History of diabetes mellitus.
   - Fasting plasma glucose ≥126 mg/dL
   - Typical symptoms of diabetes and random plasma glucose ≥200 mg/dL
   - HbA1c ≥7%
6) Alanine transaminase/aspartate aminotransferase (ALT/AST) >1.5 times the upper limit of normal or any known liver disease
7) Renal insufficiency, as defined by creatinine level >1.3 mg/dL.
8) A hemoglobin concentration < 10 gm/dL, a platelet count < 100k/mm^3 or an absolute neutrophil count < 1000/mm^3 at study entry.

19. History of myocardial infarction or cerebral vascular disease in the previous 12 months.
20. History of cognitive impairments or dementia that may impact the patient's ability to participate in the informed consent process and to appropriately complete evaluations.
21. Any current immunosuppressive therapy other than intermittent(if continuously, < 14 days)or low-dose corticosteroids (prednisolone ≤ 10 mg/day, dexamethasone ≤ 10 mg/day)
22. Current participation in any other clinical trial.
23. Participation within the previous 6 months in any clinical trial of a drug by ocular or systemic administration.
24. If female, pregnancy or lactation.
25. Any other medical condition, which, in the investigator's judgment, will interfere with the patient's ability to comply with the protocol, compromises patient safety, or interferes with the interpretation of the study results.

Inclusion/Exclusion Criteria for the SMD Study, Related to Experimental Procedures Section.

Inclusion Criteria:

1. Adult male or female over 20 years of age.
2. Clinical diagnosis of advanced SMD.
3. Visual acuity (BCVA) of the eye to receive the transplant will be no better than 20/400.
4. Visual acuity (BCVA) of the eye that is NOT to receive the transplant no better than 20/400.
5. Peripheral visual field constriction documented on standard visual field testing.
6. Electrophysiological findings consistent with SMD.
7. Medically suitable to undergo vitrectomy and subretinal injection.
8. Medically suitable for general anesthesia.
9. If female and of childbearing potential, willing to use medically effective forms of birth control during the study.

   *Medically effective methods of contraception : condoms, continuous use oral contraceptives for more than 3 months, injection or insertion of contraceptives, insertion of an intrauterine device (IUD)
10. If male, willing to use medically effective contraception during the study.
11. Willing to defer all future blood, blood component or tissue donations.
12. Able to understand and willing to sign the informed consent form.

Exclusion Criteria:

1. History of malignancy.
2. History of myocardial infarction or cerebrovascular disease in the previous 12 months.
3. Medically not suitable for transplantation of an embryonic stem cell line: Any laboratory value that falls slightly outside of the normal range will be reviewed by the medical monitor and investigators to determine its clinical significance.
   1) History of drug abuse
   2) Positive human immunodeficiency virus (HIV), hepatitis B (HBV), and hepatitis C (HCV) serologies.
   3) Any immunodeficiency.
   4) Positive cancer screening test

- Medical history & physical examination
- Chest X-ray
- Complete blood count (CBC)
- Routine U/A
- Thyroid examination (T3, T4, TSH, thyroid ultrasonography)
- If male, testicular examination
  (scrotal ultrasonography , LDH, beta-HCG)
- If male older than 40 years, digital rectal examination (DRE) and prostate-specific antigen (PSA) testing
- Stomach cancer examination (upper gastrointestinal series or esophagogastroduodenoscopy)
- Liver cancer examination (abdomen ultrasonography+ α-fetoprotein testing)
- Colorectal cancer examination (occult blood in feces + colonoscopy or colon study)
- Breast cancer examination (mammography, breast ultrasonography, breast physical examination)
- Uterine cervical cancer examination (sonohysterography, Pap Smear, pelvis examination)

5) History of diabetes mellitus
   -Fasting plasma glucose ≥126 mg/dL
   - Typical symptoms of diabetes and random plasma glucose
   ≥200 mg/dL
   -HbA1c ≥7%
6) Alanine transaminase/aspartate aminotransferase (ALT/AST) >1.5 times the upper limit of normal or any known liver disease
7) Renal insufficiency, as defined by a creatinine level >1.3 mg/dL.
8) A hemoglobin concentration <10 gm/dL, a platelet count <100 k/mm3 or an absolute neutrophil count <1000/mm3 at study entry.

4. Any current immunosuppressive therapy other than intermittent (if continuously <14 days) or low dose corticosteroids (prednisolone ≤ 10 mg/day, dexamethasone ≤ 10mg/day)
5. Current participation in any other clinical trial.
6. Participation within the previous 6 months in any clinical trial of a drug by ocular or systemic administration.

7. Any other sight-threatening ocular disease
8. Any chronic ocular medications.
9. Any history of retinal vascular disease (compromised blood-retinal barrier.)
10. Glaucoma.
11. Uveitis or other intraocular inflammatory disease.
12. Significant lens opacities or other media opacity
13. Ocular lens removal within the previous 3 months.
14. If female, pregnant or lactating
15. Any other medical condition that, in the investigator's judgment, will interfere with the patient's ability to comply with the protocol, compromises patient safety, or interferes with the interpretation of the study results.
