# Supplementary material for: Treatment of Macular Degeneration Using Embryonic Stem Cell-Derived Retinal Pigment Epithelium: Preliminary Results in Asian Patients
Source: Stem Cell Reports. 2015 Apr 30;4(5):860–72. doi: 10.1016/j.stemcr.2015.04.005 (PMC4437471; doi:10.1016/j.stemcr.2015.04.005)
Supplement: Document S2. Article plus Supplemental Information [file mmc2.pdf]

Stem Cell Reports
Article
OPEN ACCESS

Treatment of Macular Degeneration Using Embryonic Stem Cell-Derived Retinal Pigment Epithelium: Preliminary Results in Asian Patients

Won Kyung Song,[1,*] Kyung-Mi Park,[2] Hyun-Ju Kim,[2] Jae Ho Lee,[3] Jinjung Choi,[4] So Young Chong,[5] Sung Han Shim,[6] Lucian V. Del Priore,[7] and Robert Lanza[8,*]
[1]Department of Ophthalmology, CHA Bundang Medical Center, CHA University, Seongnam-si, Gyeonggi-do 463-712, Republic of Korea
[2]Development Division, CHA Biotech Co., Ltd., Seoul 135-907, Republic of Korea
[3]CHA Stem Cell Institute, CHA Biotech Co., Ltd., Seoul 135-907, Republic of Korea
[4]Division of Rheumatology, Department of Internal Medicine, CHA Bundang Medical Center, CHA University, Seongnam-si, Gyeonggi-do 463-712, Republic of Korea
[5]Division of Hematology-Oncology, Department of Internal Medicine, CHA Bundang Medical Center, CHA University, Seongnam-si, Gyeonggi-do 463-712, Republic of Korea
[6]Department of Biomedical Science, CHA University, Seoul 135-081, Republic of Korea
[7]Albert Florens Storm Eye Institute, Medical University of South Carolina, Charleston, SC 29425, USA
[8]Ocata Therapeutics, Marlborough, MA 01752, USA
*Correspondence: songwkmd@daum.net (W.K.S.), rlanza@ocata.com (R.L.)
http://dx.doi.org/10.1016/j.stemcr.2015.04.005
This is an open access article under the CC BY license (http://creativecommons.org/licenses/by/4.0/).

SUMMARY

Embryonic stem cells hold great promise for various diseases because of their unlimited capacity for self-renewal and ability to differentiate into any cell type in the body. However, despite over 3 decades of research, there have been no reports on the safety and potential efficacy of pluripotent stem cell progeny in Asian patients with any disease. Here, we report the safety and tolerability of subretinal transplantation of human embryonic-stem-cell (hESC)-derived retinal pigment epithelium in four Asian patients: two with dry age-related macular degeneration and two with Stargardt macular dystrophy. They were followed for 1 year. There was no evidence of adverse proliferation, tumorigenicity, ectopic tissue formation, or other serious safety issues related to the transplanted cells. Visual acuity improved 9–19 letters in three patients and remained stable (+1 letter) in one patient. The results confirmed that hESC-derived cells could serve as a potentially safe new source for regenerative medicine.

INTRODUCTION

Since their discovery and isolation in 1998, human embryonic stem cells (hESCs) have been considered a potentially valuable tool for generating replacement cells for therapeutic purposes (Lanza et al., 2009). However, despite success in numerous animal models, fears over tumorigenicity and immunogenicity, coupled with ethical concerns, and inefficiencies in differentiation methods have all contributed to delays in carrying out human clinical trials. Only one group has reported the results of the safety and possible biological activity of embryonic stem cell progeny in individuals with any disease (Schwartz et al., 2015), but these investigators only enrolled patients who were mostly Caucasian. Here, we confirmed the potential safety and efficacy of hESC-derived cells in Asian patients.

Loss of the retinal pigment epithelium (RPE) is an important part of the disease process in several retinal disorders, including age-related macular degeneration (AMD) and Stargardt disease. AMD is a degenerative disease that is the leading cause of visual impairment in developed countries, with the dry (nonexudative) form of AMD accounting for 85% to 90% of cases (Age-Related Eye Disease Study Research Group, 2001). Concurrent RPE and choriocapillaris atrophy are present in severe, atrophic dry AMD, with RPE atrophy preceding choriocapillaris atrophy (Schatz and McDonald, 1989; Korte et al., 1984; Leonard et al., 1997). Stargardt macular dystrophy (SMD) is the most common form of juvenile macular degeneration that is due to the production of defective rim proteins encoded by the *ABCA4* gene, leading to the accumulation of di-retinoid-pyridinium ethanolamine (A2E) in the RPE, RPE cell loss, and photoreceptor death (Glazer and Dryja, 2002). There are no known effective treatments to prevent or reverse visual loss for either disease. Since RPE loss is implicated in the pathophysiology of both disorders, RPE replacement has been suggested as a therapeutic intervention for these conditions.

Proper functioning of the RPE is important for maintaining the health and integrity of the outer retina, photoreceptors, and choriocapillaris. Healthy RPE cells play many crucial roles in the retina, including transportation of nutrients such as glucose or vitamin A from blood to the photoreceptors, secretion of growth factors, phagocytosis of the outer segments of the photoreceptors, formation of the blood-retina barrier by tight junctions, and establishment of immune privilege of the eye (Strauss, 2005; Wimmers et al., 2007). Based on the central role of RPE in the pathophysiology of AMD, researchers have attempted allogeneic and autologous RPE cell transplantations for cases of wet AMD (Binder et al., 2002; van Meurs et al., 2004; Algvere et al., 1994) and dry AMD (Algvere et al., 1997, 1999;

Joussen et al., 2007). However, most of these clinical trials have failed to show functional improvements in macular degeneration patients, possibly because of immune rejection and graft failure.

Animal studies have shown that hESC-derived RPE cell transplantation can rescue photoreceptors, resulting in the improvement of visual functions in RPE-oriented retinal degeneration models (Lund et al., 2006; Lu et al., 2009). Clinical trials of hESC-derived RPE cell transplantation have begun recently in the United States and Europe, and Schwartz et al. have reported preliminary safety data on one dry AMD patient and one SMD patient (Schwartz et al., 2012), as well as follow-up data with nine dry AMD and nine SMD patients (Schwartz et al., 2015). The patient population studied in this paper was all Caucasian, except for one African American patient with SMD. Our report provides interim results of the first pluripotent stem cell trials performed in Asian patients, who may carry different risk alleles for the development of some retinal disorders such as AMD. For example, the *Y402H* and *R80G* (in the *C3* gene) variants have been associated with AMD in Caucasians but not in Asians (Chen et al., 2006; Mori et al., 2007; Kim et al., 2008; Ng et al., 2008; Lee et al., 2008; Kondo et al., 2009; Goto et al., 2009; Pei et al., 2009). Herein, we report on four Asian patients with macular degeneration (two with AMD and two with SMD) who underwent subretinal transplantation of hESC-derived RPE and were followed for 1 year to assess safety and tolerability.

RESULTS

Derivation of RPE Cells from hESCs

The hESC-derived RPE displayed typical RPE behavior, such as pigmentation during differentiation and maturation, and also exhibited a cuboidal epithelial morphology in tissue culture. During culture, we observed clusters of pigmented RPE cell monolayers that exhibited their unique cobblestone morphology at the edges of clusters (Figures 1A and 1B). Karyotype results using g-banding showed 46XX, a normal female karyotype (Figure 1C). Thawed cells were cultured for 2–3 weeks until fully differentiated to human RPE (hRPE) cells with medium pigmentation (Figure 1D) and were stained for hRPE markers, including ZO1, PAX-6, MITF, and Bestrophin (Figures 1E–1I). We observed that >99% of cells expressed hRPE markers. For cell function analysis, we used phagocytosis assay kits using fluorescence-labeled bioparticles. Visual imaging of the differentiated hESC-derived RPE cells with fluorescence microscopy showed that most hRPE cells could phagocytize the fluorescently labeled beads (Figures 1J–1L). As for the quantification of the potency assay, fluorescence-activated cell sorting (FACS) analysis was conducted with hESC-derived RPE cells immediately post-thawing, which is more relevant to the phenotype of the cells that are actually transplanted (Figure S1). The percentage of cells phagocytized with fluorescence-labeled bioparticles was measured compared to a negative isotype control and an untreated negative control (test group at 37°C: 98.47% ± 0.32%, n = 3; isotype contol at 4°C: 34.47% ± 3.67%, n = 3; untreated negative control at 37°C: 5.52% ± 0.72%, n = 3) (Figure S1). 16-STR (short tandem repeat) genetic analysis using amplified genomic DNA (gDNA) proved that RPE cells originated from MA09. Immunostaining of OCT-4 and NANOG was conducted for impurity testing to confirm that no hESCs were present (Figure 1M). We counted DAPI-stained cells in three different fields and calculated the total cell number, and we did not see any cells that stained positive for OCT-4$^+$ or NANOG$^+$ within 21-mm dishes (Figure 1M). Additionally, we performed FACS using fluorescent labels for OCT-4 and TRA-1-60 and demonstrated no contamination by hESCs in the final product (PRE-0008) when 10,000 cells were analyzed for each marker: OCT-4, 0.28%; TRA-1-60, 0.02% (positive control: OCT-4, 53.26%; TRA-1-60, 40.96% [hES-MA09 cells were maintained on mouse embryonic fibroblast feeder cells]; negative control [NPC, neural precursor cells]: OCT-4, 0.47%; TRA-1-60, 0.35%) (Figure 1N). On further safety analysis through quality control testing, we confirmed the pathogen- and virus-free status of clinical samples by sterility, mycoplasma, and endotoxin detection following the Korean Pharmacopoeia and the Ministry of Food and Drug Safety (MFDS) guidelines for pathogen and virus testing. For the clinical studies, we transplanted >90% of viable cells after their final formulation in BSS Plus solution.

Clinical Trial Results

The first advanced dry-AMD patient was a 79-year-old male with an initial best-corrected visual acuity (BCVA) of the study eye of one letter read and of the fellow eye of 20/25 (80 letters) on a Bailey-Lovie chart. During surgery, retinal detachment was difficult to initiate at the first retinotomy site, and subretinal cells were injected at a second site. A small subretinal hemorrhage was noted at this second site (Figure 2B). We estimated that 4×10^4 cells were injected subretinally. The hemorrhage absorbed spontaneously at postoperative 26 weeks (Figure 2C). Immunosuppression was stopped 4 weeks postoperatively because of repeated elevation of serum creatinine levels, blood urea nitrogen (BUN) levels, and potassium levels, as well as bone marrow suppression and diarrhea; these adverse events returned to preoperative levels after the cessation of immunosuppression. An epiretinal membrane developed at 2 weeks, with dark brown pre-retinal pigmentation from 3 weeks. The epiretinal membrane enlarged until 8 weeks, causing minimal distortion of the underlying inner retina, and the pre-retinal

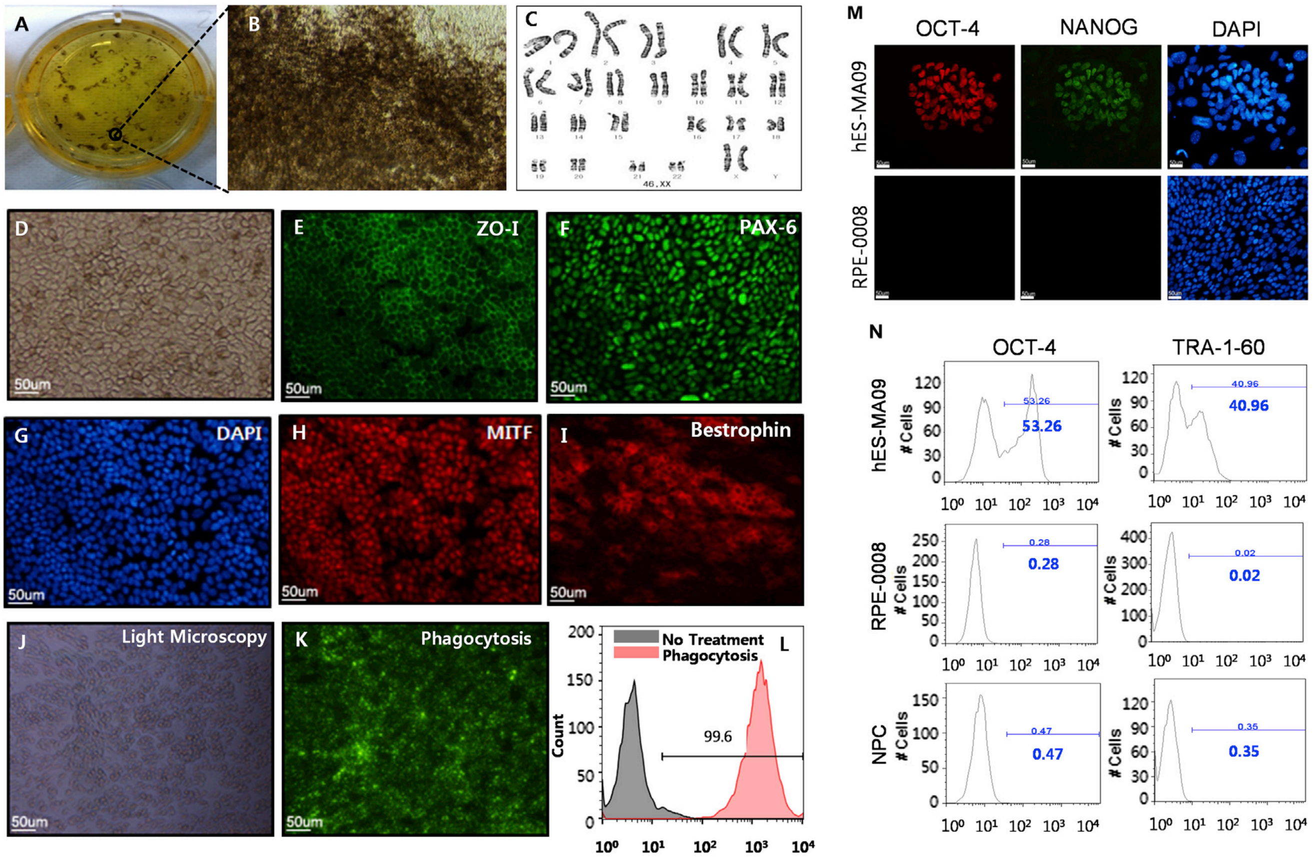

Figure 1. Characterization of the Clinical Product: Identity, Potency, and Purity

(A) RPE clusters were obtained by culturing an embryoid body attached to a six-well plate for about 8 weeks.
(B) The cells at the edge of the pigmented cluster displayed typical morphology of hRPE with hypo-pigmentation of the leading edge.
(C) A normal female karyotype (46XX) is shown.
(D) A confluent cobblestone monolayer was observed via Hoffman modulation contrast microscopy.
(E and F) Cells were positive for ZO-1 (E) and PAX-6 (F).
(G and H) DAPI staining in (G) was used to identify the location of the nuclei corresponding to ZO-1 (in E) and MITF (H) at the same time. ZO-1 and MITF were double stained in one sample.
(I) Mature RPE cells were recognized with anti-Bestrophin.
(J–L) Phagocytosis assay results were shown. Fluorescence microscopy image and FACS analyses of the differentiated hESC-derived RPE cells demonstrate that most of the cells (99.6%) were phagocytized with the fluorescent-labeled particles.
(M and N) Purity was assessed by the absence of hESCs of the final product by immunocytochemical staining for OCT-4 and NANOG (M) and FACS analysis demonstrating the absence of OCT-4 and TRA-1-60 (N).
Scale bars, 50 μm.

pigmentation area increased and darkened by 13 weeks, with no change through 1 year (Figure 2D). Subretinal pigmentation started at 3 weeks as an oval-shaped, localized black clump. The clump was slightly increased in size until 13 weeks as the subretinal hemorrhage decreased. Blocked autofluorescence was noted at these pre-retinal and subretinal pigmentation areas (Figure 2F). Choroidal neovascularization (CNV) was present on fluorescein angiography temporal to the area of preoperative geographic atrophy (GA) at postoperative 33 weeks (Figures 2E and 2F). Leakage on fluorescein angiography improved (Figure 2G) after three monthly intravitreal Lucentis (0.5 mg/0.05 ml, Genentech) injections. At the 1-year visit, BCVA was stable in the study eye (two letters read; Table 1) without subjective symptoms, and an epiretinal membrane persisted with minimal retinal puckering (Figure 2H). There was minimal enlargement of the central scotoma on Goldmann perimetry and no significant change in electroretinography (ERG) and multifocal electroretinography (mfERG) (Figures 2J and 2L; Table 2). The BCVA of the fellow eye was 20/32 (75 letters). Coryza, senile purpura, gynecomastia, constipation, and allergic conjunctivitis were adverse events with no causal

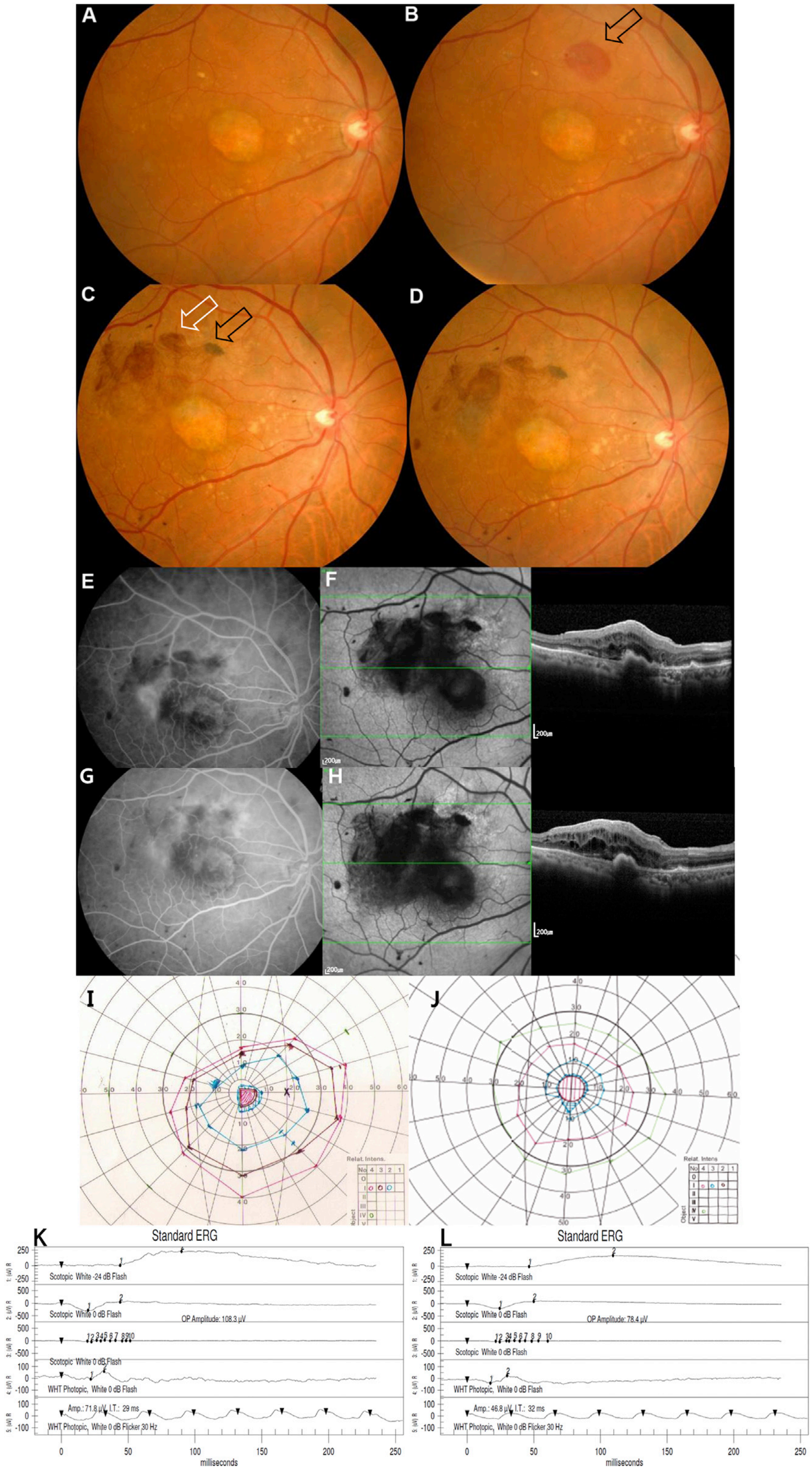

Figure 2. Ophthalmologic Results of the First Dry AMD Patient

(A) Baseline fundus photography with geographic atrophy and drusens.

(B) Small subretinal hemorrhage at post-operative day 1 at the nasal injection site (arrow).

(C) Fundus photography at post-operative 26 weeks showing absorption of hemorrhage. Subretinal pigment is present at the nasal injection site (black arrow) and pre-retinal pigmentation and epiretinal membrane are visible superotemporal to the fovea (white arrow).

(D and E) Fundus photography (D) and fluorescence angiography (E) at post-operative 33 weeks reveal a neovascular membrane temporal to the fovea.

(F) Autofluorescence imaging shows wide-spread hypo-autofluorescence (left); OCT demonstrates sub-RPE elevation and sub-retinal and intraretinal fluid (right).

(G) The CNV is less active on fluorescein angiography at post-operative 52 weeks after three monthly intravitreal Lucentis treatments.

(H) There is no significant change in auto-fluorescence and OCT.

(I and J) There is minimal enlargement of central scotoma (J) compared with baseline (I) based on Goldmann visual field examination.

(K and L) Electroretinography at baseline (K) and at the 1-year visit (L) showed no significant changes.

relationship with the study procedure. The patient completed a 1-year follow-up with no ocular or systemic serious adverse events.

The second dry AMD patient was a 65-year-old male with an initial BCVA of 20/320 (25 Early Treatment Diabetic Retinopathy Study [ETDRS] letters) in the study eye and

Table 1. Visual Acuity Changes of the Study Eye of Macular Degeneration Patients after hESC-RPE Cell Injection

	AMD Patient 1		AMD Patient 2		SMD Patient 1		SMD Patient 2	
Timeline	BCVA	ETDRS (Number of Letters)	BCVA	ETDRS (Number of Letters)	BCVA	ETDRS (Number of Letters)	BCVA	ETDRS (Number of Letters)
Baseline	CF4ft	1	20/320	25	CF2ft	1	20/640	13
1 week	CF4ft	2	20/320	27	CF2ft	0	20/500	18
2 weeks	CF4ft	4	20/320	28	CF2ft	0	20/500	18
3 weeks	CF4ft	3	20/250	30	CF2ft	5	20/400	22
4 weeks	CF4ft	3	20/250	33	CF2ft	5	20/250	29
6 weeks	CF4ft	2	20/250	33	20/800	8	20/250	31
8 weeks	CF4ft	2	20/200	35	20/640	10	20/200	32
13 weeks	CF4ft	2	20/250	33	20/800	10	20/200	34
26 weeks	CF4ft	3	20/250	35	20/640	12	20/250	35
39 weeks	CF4ft	3	20/200	34	20/500	14	20/200	33
52 weeks	CF4ft	2	20/200	34	20/640	13	20/250	32

CF4ft, counting fingers at 4 ft; CF2ft, counting fingers at 2 ft.

20/80 (55 letters) in the fellow eye. During subretinal injection of cells through the first retinotomy at the inferotemporal macula, the bleb encroached upon the fovea. As per the protocol, the injection was stopped, and another retinotomy and subretinal injection of the remaining cells were performed through a distant retinotomy in the superotemporal macula. Intraocular pressure elevation, corneal erosions, and corneal abrasion were adverse events related to the vitrectomy procedure that subsided after the administration of topical eye drops. Subretinal pigmentation was observed starting at postoperative 3 weeks as dark brown to black scattered small oval clumps inside the bleb area, and the number of clumps increased until 6 weeks and did not change through 52 weeks (Figures 3A and 3B). After the surgery, stippled hyper-autofluorescence dots were observed at the border of the atrophic zone where the bleb was

Table 2. mfERG Amplitude Changes of Hexagons Including the hESC-RPE-Injected Bleb Site of the Study Eyes Compared with the Corresponding Area of the Fellow Eye

	Study Eyes			Fellow Eyes		
Timeline	Mean ± SD[a]	Median[a]	p[b]	Mean ± SD[a]	Median[a]	p[c]
Baseline	9.74 ± 5.22	11.90	NA	10.85 ± 4.32	12.41	0.5309
3 months	12.27 ± 4.79	13.26	NA	14.29 ± 4.67	16.84	0.4034
6 months	12.43 ± 5.33	13.61	NA	11.61 ± 4.84	13.93	0.8345
12 months	13.66 ± 7.68	17.90	NA	14.66 ± 7.11	19.23	0.5309
3 months-baseline[d]	2.52 ± 2.28	1.93	0.1250	3.44 ± 1.48	3.73	0.5309
6 months-baseline[d]	2.68 ± 3.27	3.67	0.1875	0.76 ± 1.42	0.63	0.2963
12 months-baseline[d]	3.92 ± 3.64	1.89	0.0625	3.81 ± 3.13	4.42	0.8345

Hexagons in which the bleb involved at least half of its area were included. For both study eyes and fellow eyes, n = 5; a total of four eyes and five blebs due to two blebs in the second AMD patient. NA, not applicable.
[a]Response density (nanovolts per square degree).
[b]Wilcoxon's signed-rank test of the changes of the study eyes.
[c]Wilcoxon's rank-sum test between the changes in the study eyes and those in the fellow eyes.
[d]3 months(6 months, 12 months)-baseline indicates the change from baseline to the corresponding month after surgery.

involved (Figures 3C and 3D). An epiretinal membrane developed at postoperative 2 weeks, with pre-retinal pigmentation starting at 3 weeks, which increased in size until 15 weeks. Minimal puckering of the underlying retina accompanied this pigmented epiretinal membrane. Stippled hypo-autofluorescence at subretinal pigmented areas and patchy hypo-autofluorescence at preretinal pigmentation areas were noted (Figure 3D). Retinal cysts were noted at the center of the GA area at 26 weeks on spectral domain optical coherence tomography (SD-OCT). There were focal pinpoint areas of hyperfluorescence on the angiogram at 26 weeks, with no definite change through 52 weeks. There was no sign of intraocular inflammation such as anterior chamber cells or vitreous cells. At the postoperative 1-year visit, BCVA had gradually improved to 20/200 (34 ETDRS letters; Table 1) in the study eye. The central scotoma was observed to diminish in intensity via Goldmann perimetry (Figures 3E and 3F). ERG (Figures 3G and 3H) and mfERG of the injection sites (Table 2) were stable after the surgery. In the fellow eye, BCVA deteriorated to 20/200 (35 letters). Systemic adverse events considered to be unrelated to the study procedures included laryngopharyngeal reflux that developed at 3 weeks and improved at 13 weeks, upper respiratory infection with rhinorrhea at 42 weeks, and potassium level elevation at 52 weeks. These adverse events improved after medical therapy. Diarrhea, indigestion, and tinnitus were mild events not related to the study procedures and subsided spontaneously. The patient experienced intermittent right-hand tremor starting at approximately 15 weeks and received acupuncture several times without informing the investigators. At 26 weeks, a neurologist evaluated the patient and determined this event as age-related changes not related to the study procedure. Pneumonia, which may have been related to immunosuppression, was diagnosed at 8 weeks at our pulmonary department and then subsided after 3 days of oral antibiotics treatment.

Our first SMD patient was a 45-year-old male with an initial BCVA of counting fingers (one ETDRS letter). There were no signs of anterior chamber or vitreous cell or flare beyond what would be typically seen in the postoperative period. The patient had corneal erosions in his left eye after the ERG, and he recovered without sequelae with prophylactic topical antibiotic eye drops. No pigmentation or autofluorescence changes were noted after the hESC-RPE transplantation in this patient (Figures 4A–4D). At postoperative 52 weeks, his BCVA had gradually improved to 20/640 (13 ETDRS letters; Table 1), and a smaller central scotoma was observed via Goldmann visual field examination (Figures 4E and 4F). BCVA of the fellow eye was 20/800 (4 ETDRS letters read) before the surgery and 20/500 (13 letters) at 52 weeks. There was no change in the anatomic appearance on SD-OCT and no change in either the ERG (Figures 4D and 4H) or mfERG (Table 2) after surgery. Herpetic vesicles developed on the patient's right arm that were possibly related to immunosuppressive mycophenolate mofetil (MMF); these were treated with topical acyclovir application without changes in his immunosuppressive regimen. Skin bullae at the left forearm, a contusion of the right hand, external otitis, rhinorrhea, sneezing, fatigue, headache, upper respiratory infection, and chronic gingivitis were mild adverse events observed that had no causal relationship with the procedure, as determined by the rheumatologist and relevant specialists.

The second SMD patient was a 40-year-old male. His BCVA improved from 20/640 (13 ETDRS letters) to 20/250 (32 letters) at 1 year. BCVA of the fellow eye was 20/250 (32 letters) at baseline and 20/160 (41 letters) at 1 year. Subretinal pigmentation started at 4 weeks in the bleb area, and the number of pigmentations increased in number until 6 weeks, with hypo-autofluorescence that persisted until 52 weeks (Figures 5A–5D). Multiple increased autofluorescence spots were also observed inside the bleb area where hESC-RPE was injected (Figure 5D). Because of a large initial central scotoma and poor visual function, visual field examinations were unreliable, and there was no obvious change after surgery. ERG and the multifocal ERG of the injection site were stable throughout 52 weeks (Figures 5E and 5F; Table 2). Upper respiratory infection, aggravation of reflux esophagitis, and loss of a dental implant were mild adverse events considered unrelated to the study procedures.

The first SMD patient had three missense mutations: c.983A > T (Glu328Val), c.1933G > A (Asp645Asn), and c.3106G > A (Glu1036Lys). The second SMD patient had two missense mutations: c.2894A > G (Asn965Ser) and c.4972A > C (Ser1658Arg). All of these mutations, except for the c.4972A > C mutation in S-004, were previously reported as Stargardt-associated mutations (Jaakson et al., 2003).

DISCUSSION

The purpose of this study was to determine the safety and tolerability of the subretinal injection of hESC-derived RPE cells as a treatment for dry AMD and SMD. No serious systemic or ocular adverse events occurred in these four Asian patients. Ophthalmological examinations, including ETDRS visual acuity, visual field examination, color fundus photography, fundus fluorescein angiography, optical coherence tomography, fundus autofluorescence photography, ERG, and mfERG did not identify any significant safety concerns after surgery.

This present study was an open-label trial of patients who had poor visual function preoperatively with a large central

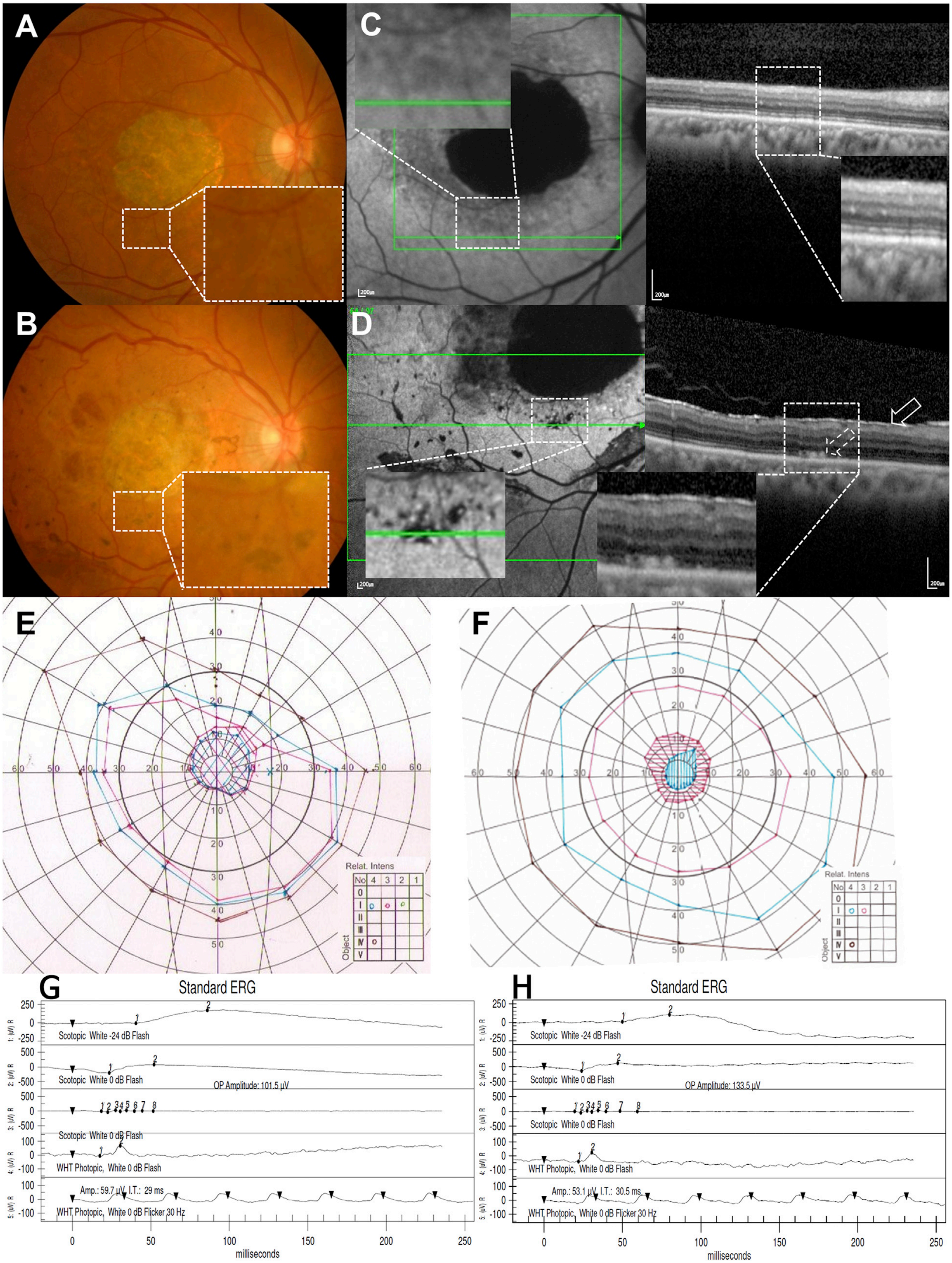

(legend on next page)

scotoma, which hindered our ability to perform reliable measurements of visual functions. In the two dry-AMD patients, visual acuity in the treated eyes improved by one letter (stable at counting fingers at 4 ft) and nine letters (a two-line improvement from 20/320 to 20/200) at 52 weeks, respectively. In contrast, the fellow (untreated) eyes decreased by 6 and 20 letters, respectively, during the same time period. In the two SMD patients, visual acuity improved in the treated eyes by 12 letters (counting fingers at 2 ft to 20/640) and 19 letters (a four-line improvement from 20/640 to 20/250), respectively, compared with nine letters of improvement in the fellow eyes at 52 weeks compared to baseline. The visual acuity improvement noted in the fellow eyes of SMD patients may be due to poorer baseline visual acuity than in the fellow eyes of the dry AMD patients. A 15-letter improvement (a doubling of the visual angle) is generally accepted as a clinically significant change. The sample size in these studies (SMD phase 1: 3 patients; dry AMD phase 1/2a: total of 12 patients, with 3 for each dose) was not powered to detect an improvement in visual acuity. Thus, the visual acuity measurement in these preliminary data should be primarily interpreted as a safety parameter to monitor for adverse effects from the transplants. It is important to note that there was no decline in visual acuity in any of the four eyes.

Humphrey visual field examinations were unreliable because of poor fixation and high false-negatives in nearly all of the participants. Goldmann visual field (GVF) examinations were assessed in these patients. There was no significant change in the visual field and mfERG examinations in these eyes.

In the two dry AMD patients and one SMD patient, increasing subretinal pigmentation developed at the hESC-RPE cell injection site as has been noted previously (Schwartz et al., 2012, 2015). In the second dry AMD patient and the second SMD patient, multiple spots of increased autofluorescence were also observed at the injection sites. It is tempting to speculate that the subretinal pigmentation may represent engrafted RPE, and this conclusion is strengthened by the observations of small deposits on the inner aspects of Bruch's membrane after surgery on OCT. However, it is known that pigment is not a robust marker of donor cells, since ingestion of donor pigment by host macrophages may produce a similar ophthalmoscopic appearance. Although there is no host RPE in the regions of atrophy, prior studies have shown that native adult RPE cells may proliferate and migrate within 72 hr after retinal detachment in cats (Anderson et al., 1981); migration of host RPE and ingestion of pigment by these cells may also explain the appearance of subretinal pigment after hESC-RPE injection. It is important to note that the subretinal pigmentation and stippled hyper-autofluorescence spots noted after subretinal hESC-RPE injection have not been reported in eyes after similar procedures with Ca- and Mg-free solution in other trials where transient retinal detachment was performed in order to achieve subretinal viral vector administration (Maguire et al., 2008).

One of the largest concerns in the clinical applications of hESC-derived cell therapy is potential tumorigenicity. Teratoma formation is observed within 8 weeks (Klimanskaya et al., 2005). All of our patients were followed for at least 1 year, and, using color fundus photography, autofluorescence imaging, and SD-OCT with 3-μm resolution, we found no unwanted abnormal proliferation suggesting teratoma formation.

Since the donor hESC-derived cells are allografts, there is the possibility of graft rejection after transplantation. Previous allogeneic RPE cell transplantations in dry AMD resulted in graft rejections in some cases, although it started later in the treatment course and was seen less frequently than in patients with wet AMD (Algvere et al., 1997, 1999). We did not observe any clinically significant intraocular inflammation and detected no signs of obvious immune rejection including fluid collection, edema, fibrous membrane formation, persistent leakage on fluorescein angiography, or graying or loss of pigmentation of the graft in patients who underwent graft transplantation and were able to maintain systemic immunosuppression. The second dry AMD patient developed intraretinal cysts and dye pooling on fluorescein angiography that did not change for 52 weeks after surgery. No significant changes in visual acuity, multifocal ERG, symptoms, or

Figure 3. Ophthalmologic Results of the Second Dry AMD Patient

(A and B) Baseline (A) and 52-week-postoperative (B) fundus photography of the second dry AMD patient, showing subretinal pigmentation (B, inset) after surgery.

(C and D) Baseline (C) and 52-week-postoperative (D) autofluorescence imaging and SD-OCT. Note the stippled hypo-autofluorescence present after surgery at the border of the atrophic zone that may represent blockage from subretinal pigmentation and stippled hyper-autofluorescence at the same area underlying the bleb (D, left, inset). Subretinal deposits (D, right, dashed arrow on OCT) are seen in the cell-transplanted areas that were not present prior to surgery. There is an epiretinal membrane present postoperatively (D, right, solid arrow). Patchy hypo-autofluorescence present at preretinal pigmentation areas (D, left).

(E and F) GVF examinations at baseline (E) and post-operative 52 weeks (F) show a central scotoma of diminished intensity.

(G and H) ERG examinations at baseline (G) and at post-operative 52 weeks (H) show no significant changes.

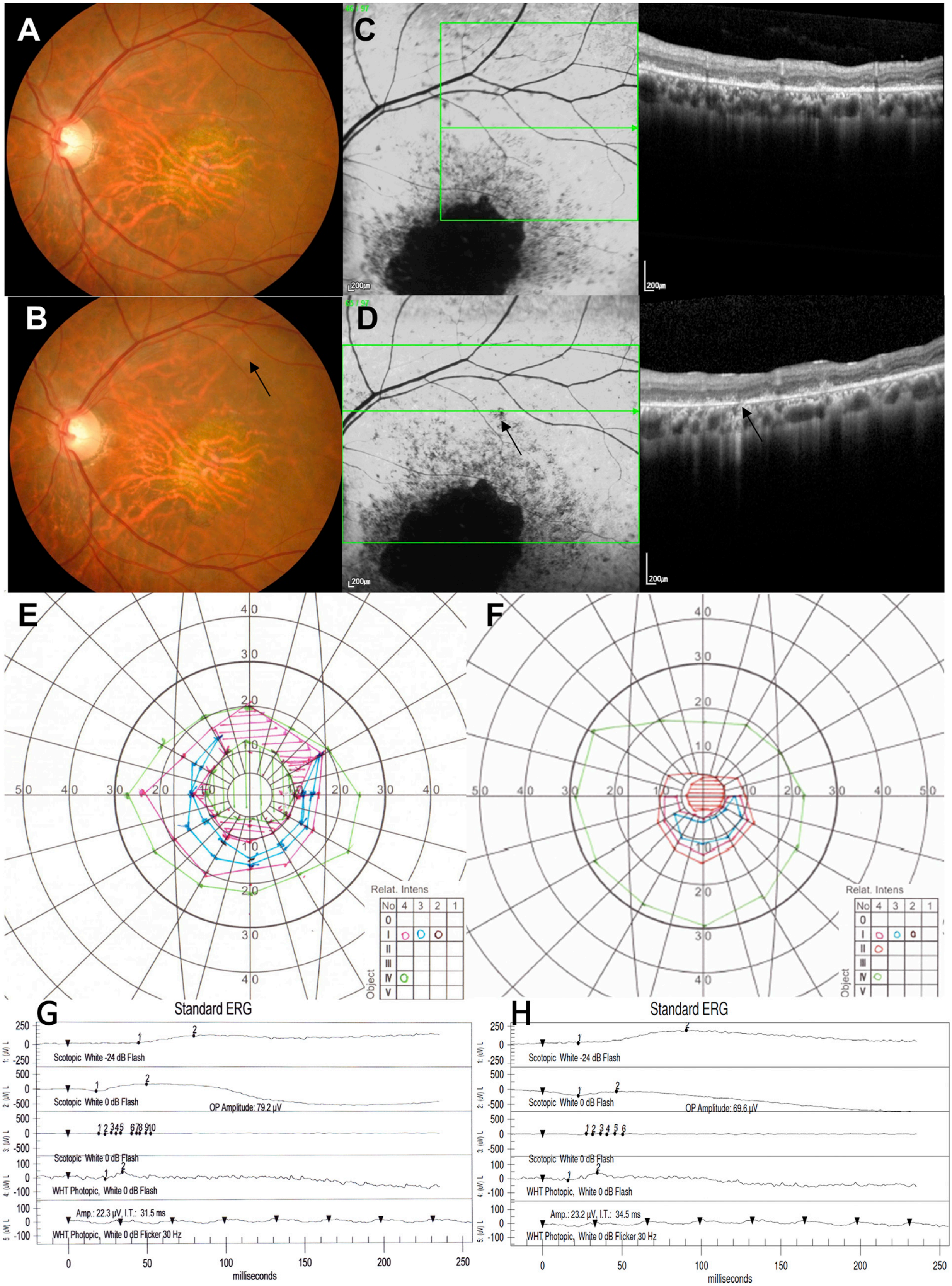

(legend on next page)

other signs of intraocular inflammation accompanied this change. The subretinal and pre-retinal pigmentations persisted without change. Cystoid macular edema is present in approximately 20%–40% of patients with epiretinal membrane (Wickham and Gregor, 2013). The retinal cyst may have accompanied the epiretinal membrane in this patient. However, this does not rule out the possibility of graft rejection.

The moderate adverse events related to immunosuppression included creatinine level elevation in the first dry AMD patient and pneumonia in the second dry AMD patient. Immunosuppressive medication was stopped in the first patient because of repeated deterioration of renal function, including creatinine and potassium level elevation. We could continue the dosage of immunosuppression per protocol in three patients. The dose and mandatory period of immunosuppression should be determined in the future trials.

The two dry AMD patients developed epiretinal membranes with pigmentation. This is relatively high compared with the rate of epiretinal membrane development after the vitrectomy procedure. It may arise from inadvertent pre-retinal injection of cells or reflux of transplanted cells from the subretinal space. These pre-retinal patches of pigmentation did not contract and cause macular wrinkling enough to require additional surgery during the follow-up interval.

The first dry AMD patient developed CNV in the hESC-RPE transplanted eye at 33 weeks. The location of the CNV was not primarily in the bleb area, and only the superior margin of the CNV adjoined the inferior margin of the previous bleb location. This patient had drusenoid subretinal accumulation with GA in the study eye and drusenoid pigment epithelial detachment (PED) in the fellow eye. A previous report showed a relatively high rate of development of CNV (23%) in eyes with drusenoid PED with no advanced AMD at baseline (Cukras et al., 2010). The CNV may have developed as a natural course, or it may have been due to trauma to Bruch's membrane during the surgical procedure or from the injected hESC-RPE.

The present report confirmed the feasibility and preliminary safety of hESC-RPE cell therapy. Continued follow-up and further study are needed to determine the long-term safety and efficacy of hESC-derived cells as a potential source of replacement cells for the treatment of macular degeneration.

EXPERIMENTAL PROCEDURES

hESC-derived RPE cells were manufactured in a fully validated good-manufacturing-practice (GMP) facility under strict environmental control monitoring systems and routine microbial testing regimens at CHA Biotech. Master and working cell banks were established using the hESC line MA09 (Ocata Therapeutics, previously Advanced Cell Technology) (Schwartz et al., 2012), which was registered with the Korea Centers for Disease Control and Prevention as an imported stem cell line. The comparability of MA09-hRPE cells manufactured from both GMP sites (CHA Biotech versus Ocata Therapeutics) was confirmed by characterizing the MA09-hRPE cells in terms of karyotype, genetic analysis, identity, purity, and potency by using real-time PCR, immunocytochemistry, FACS analysis, and phagocytosis assays (Supplemental Information).

The MFDS and CHA Bundang Medical Center institutional review board (IRB) approval was obtained to carry out two prospective clinical trials to evaluate the safety and tolerability of the cells in patients with SMD and dry AMD (registered with ClinicalTrials.gov [https://clinicaltrials.gov]; numbers NCT01625559 and NCT01674829). The protocols are similar to those of the U.S. studies (NCT01345006 and NCT01344993), with some differences regarding cancer screening and immunosuppression (see Supplemental Information). Cancer screening included complete history recording, physical examination, laboratory tests, and further ultrasonography, endoscopy, and biopsy when needed. Immunosuppression was started at lower dosages of tacrolimus and MMF; the doses were gradually titrated according to each patient's serum tacrolimus level (3–7 ng/ml), with tolerability judged by a clinical rheumatologic specialist, while the ophthalmologists monitored the patient for changes in the clinical examinations. Lower MMF dosages of 1.0–1.5 g/day were used according to previous reports regarding its safety and efficacy in the Asian organ transplantation patients (Tsang et al., 2000; Kim et al., 2010). Both immunosuppressive drugs were started 1 week prior to the surgical procedure and were continued for a period of 7 weeks until post-operative 6 weeks. Next, the tacrolimus was discontinued, and the MMF was continued for an additional 7 weeks before being slowly tapered thereafter. In the case of SMD, *ABCA4* genes were examined using the exome sequencing method, with a portion of the blood specimen archived for safety purposes at baseline. Surgeries were carried out at the CHA Bundang Medical Center by a single surgeon (W.K.S.). Posterior pars plana vitrectomy was performed in the eye with the worse vision, with induction of posterior vitreous detachment (PVD). A volume of 150 μl of RPE reconstituted in BSS Plus was injected into the subretinal space via a 38-G subretinal cannula, delivering the target dose of 5×10^4 RPE cells. Patients were kept in a supine position for at

Figure 4. Ophthalmologic Results of the First SMD Patient

(A–D) Baseline (A) and 52-week-postoperative (B) fundus photography, baseline (C), and 52-week-postoperative (D) autofluorescence imaging and SD-OCT showing no signs of immune rejection, tumor formation, or adverse event related to the surgical procedure. No obvious pigmentation (arrow indicates the cell-injected retinotomy site) was noted after hESC-RPE transplantation.

(E and F) GVF examinations at baseline (E) and post-operative 52 weeks (F) show a central scotoma of diminished size.

(G and H) ERG examinations at baseline (G) and post-operative 52 weeks (H) show no change.

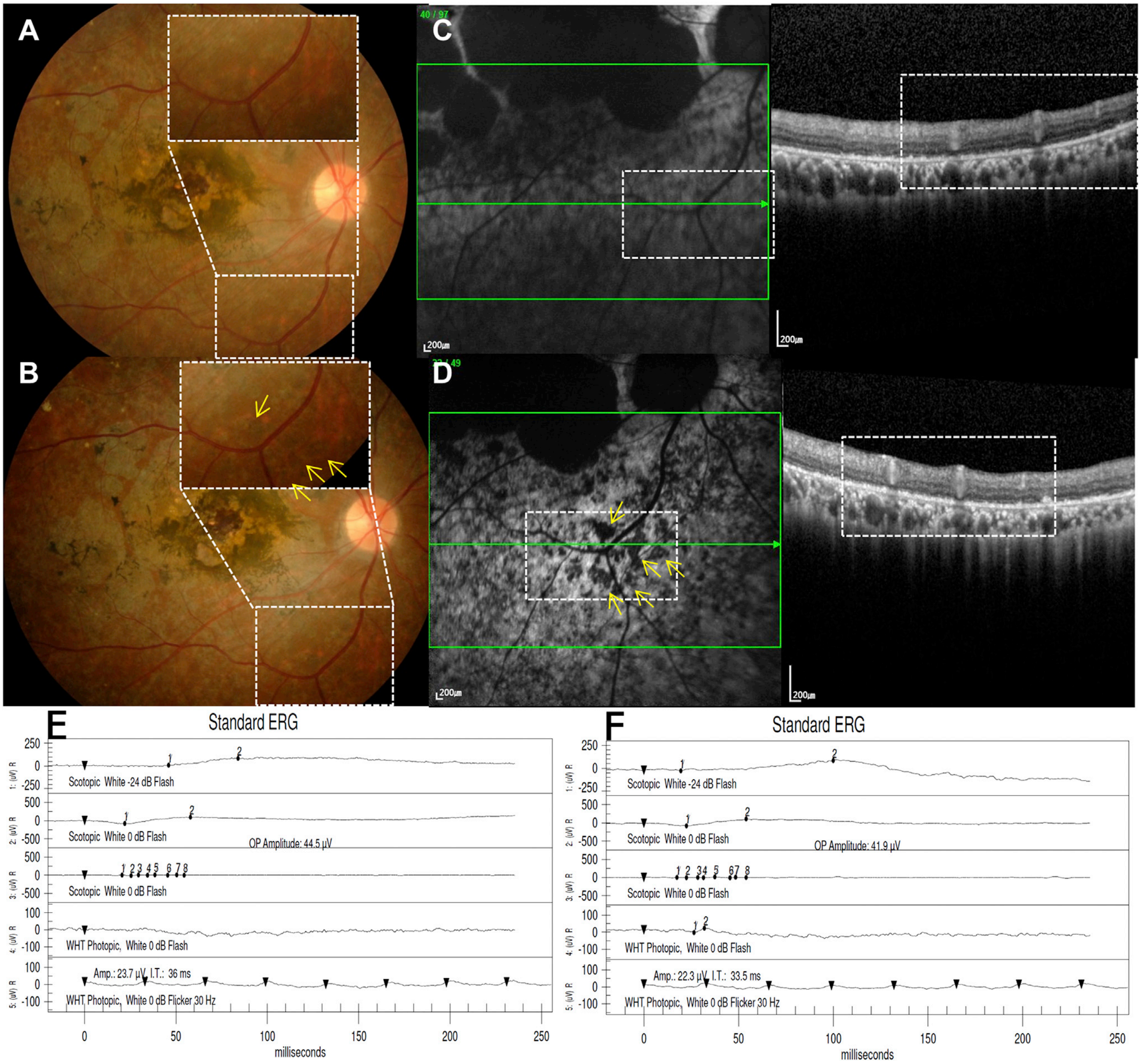

Figure 5. Ophthalmologic Results of the Second SMD Patient

(A and B) Baseline (A) and 52-week postoperative (B) fundus photography show subretinal pigmentation (B, inset, yellow arrows) in the bleb area that may represent engraftment of injected hESC-RPE cells.

(C and D) SD-OCT at baseline (C, right) and postoperative 52 weeks (D, right) at corresponding sites showing a monolayer of the RPE layer. Fundus autofluorescence images (left panels of C and D) show stippling of autofluorescence at 52 weeks after surgery (D, inset, yellow arrows).

(E and F) There is no change in the electroretinogram from baseline (E) to postoperative 52 weeks (F).

least 6 hr after the operation. Patients were closely monitored by physical examinations, laboratory examinations, and ophthalmologic examinations, including BCVA analysis using a Bailey-Lovie chart, visual field examination using Goldmann visual field testing (Projection perimeter MK-70ST L-1550, Inami Ophthalmic Instruments) and/or automated testing using a validated Humphrey perimeter (Humphrey Field Analyzer; Carl Zeiss Meditec), fundus photography, fluorescein angiography (KOWA VX-10i; Kowa), SD-OCT, fundus autofluorescence photography (Spectralis OCT; Heidelberg Engineering), and ERG (UTAS E-3000 system; LKC Technologies) (see Tables S1 and S2 for the schedule of assessments).

SUPPLEMENTAL INFORMATION

Supplemental Information includes Supplemental Experimental Procedures, one figure, and two tables and can be found with this article online at http://dx.doi.org/10.1016/j.stemcr.2015.04.005.

AUTHOR CONTRIBUTIONS

Conception and design: R.L., L.V.D.P., and W.K.S.; analysis and interpretation: W.K.S., H.-J.K., J.H.L., J.C., S.Y.C., S.H.S., L.V.D.P., and R.L.; writing the article: W.K.S. and H.-J.K.; critical revision of the article: W.K.S., R.L., and L.V.D.P.; final approval of the article: W.K.S., R.L, and L.V.D.P.; data collection: W.K.S., H.-J.K., J.H.L., J.C., S.H.S., and K.-M.P.; provision of materials, patients, or resources: W.K.S., R.L., and K.-M.P.; literature research: W.K.S., H.-J.K., and S.H.S.

ACKNOWLEDGMENTS

We express sincere gratitude to Sung Woo Ha, MD, and Kyung Soon Kim, PhD, for their efforts in the Korean protocol development and securing Korean MFDS and IRB approval; the Independent Data Monitoring Committee, Sung Chul Lee, Oh Woong Kwon, Kwang Soo Kim, and Hyonggin An; co-investigator Hee Jung Kwon for surgical assistance, co-investigator Sung Woo Choi for mfERG analysis; clinical research coordinators Myung Hee Choi, Yona Bae, Ju Eun Lee, and Su Youn Kim; and the pioneering patients and their families. This research was supported by the CHA Biotech Co., Ltd., by their grant of the Korea Health Technology R&D Project through the Korea Health Industry Development Institute (KHIDI), funded by the Ministry of Health & Welfare, Republic of Korea: grant numbers HI12C1794(A121941) and HI12C0447(A120506). The sponsor (CHA Biotech Co., Ltd.) participated in hESC-RPE manufacture and in-use-protocol process, study design, and partial report preparation. Kyung-Mi Park is an employee of CHA Biotech. Hyun Ju Kim and Jae Ho Lee are employees of CHA Biotech and have received stock options.

Received: October 16, 2014
Revised: April 3, 2015
Accepted: April 6, 2015
Published: April 30, 2015

REFERENCES

Algvere, P.V., Berglin, L., Gouras, P., and Sheng, Y. (1994). Transplantation of fetal retinal pigment epithelium in age-related macular degeneration with subfoveal neovascularization. Graefes Arch. Clin. Exp. Ophthalmol. *232*, 707–716.

Algvere, P.V., Berglin, L., Gouras, P., Sheng, Y., and Kopp, E.D. (1997). Transplantation of RPE in age-related macular degeneration: observations in disciform lesions and dry RPE atrophy. Graefes Arch. Clin. Exp. Ophthalmol. *235*, 149–158.

Algvere, P.V., Gouras, P., and Dafgård Kopp, E. (1999). Long-term outcome of RPE allografts in non-immunosuppressed patients with AMD. Eur. J. Ophthalmol. *9*, 217–230.

Anderson, D.H., Stern, W.H., Fisher, S.K., Erickson, P.A., and Borgula, G.A. (1981). The onset of pigment epithelial proliferation after retinal detachment. Invest. Ophthalmol. Vis. Sci. *21*, 10–16.

Age-Related Eye Disease Study Research Group (2001). A randomized, placebo-controlled, clinical trial of high-dose supplementation with vitamins C and E, beta carotene, and zinc for age-related macular degeneration and vision loss: AREDS report no. 8. Arch. Ophthalmol. *119*, 1417–1436.

Binder, S., Stolba, U., Krebs, I., Kellner, L., Jahn, C., Feichtinger, H., Povelka, M., Frohner, U., Kruger, A., Hilgers, R.D., and Krugluger, W. (2002). Transplantation of autologous retinal pigment epithelium in eyes with foveal neovascularization resulting from age-related macular degeneration: a pilot study. Am. J. Ophthalmol. *133*, 215–225.

Chen, L.J., Liu, D.T., Tam, P.O., Chan, W.M., Liu, K., Chong, K.K., Lam, D.S., and Pang, C.P. (2006). Association of complement factor H polymorphisms with exudative age-related macular degeneration. Mol. Vis. *12*, 1536–1542.

Cukras, C., Agrón, E., Klein, M.L., Ferris, F.L., 3rd, Chew, E.Y., Gensler, G., and Wong, W.T.; Age-Related Eye Disease Study Research Group (2010). Natural history of drusenoid pigment epithelial detachment in age-related macular degeneration: Age-Related Eye Disease Study Report No. 28. Ophthalmology *117*, 489–499.

Glazer, L.C., and Dryja, T.P. (2002). Understanding the etiology of Stargardt's disease. Ophthalmol. Clin. North Am. *15*, 93–100, viii.

Goto, A., Akahori, M., Okamoto, H., Minami, M., Terauchi, N., Haruhata, Y., Obazawa, M., Noda, T., Honda, M., Mizota, A., et al. (2009). Genetic analysis of typical wet-type age-related macular degeneration and polypoidal choroidal vasculopathy in Japanese population. J. Ocul. Biol. Dis. Infor. *2*, 164–175.

Jaakson, K., Zernant, J., Külm, M., Hutchinson, A., Tonisson, N., Glavac, D., Ravnik-Glavac, M., Hawlina, M., Meltzer, M.R., Caruso, R.C., et al. (2003). Genotyping microarray (gene chip) for the ABCR (ABCA4) gene. Hum. Mutat. *22*, 395–403.

Joussen, A.M., Joeres, S., Fawzy, N., Heussen, F.M., Llacer, H., van Meurs, J.C., and Kirchhof, B. (2007). Autologous translocation of the choroid and retinal pigment epithelium in patients with geographic atrophy. Ophthalmology *114*, 551–560.

Kim, K., Lee, S., Hwang, S., Kim, K., Ahn, C., Moon, D., Ha, T., Song, G., Jung, D., Choi, N., et al. (2010). Does calcineurin inhibitor plus mycophenolate mofetil combination therapy decrease the risk of late acute rejection after liver transplantation? J. Korean Soc. Transplant. *24*, 93–100.

Kim, N.R., Kang, J.H., Kwon, O.W., Lee, S.J., Oh, J.H., and Chin, H.S. (2008). Association between complement factor H gene polymorphisms and neovascular age-related macular degeneration in Koreans. Invest. Ophthalmol. Vis. Sci. *49*, 2071–2076.

Klimanskaya, I., Chung, Y., Meisner, L., Johnson, J., West, M.D., and Lanza, R. (2005). Human embryonic stem cells derived without feeder cells. Lancet *365*, 1636–1641.

Kondo, N., Honda, S., Kuno, S., and Negi, A. (2009). Coding variant I62V in the complement factor H gene is strongly associated with polypoidal choroidal vasculopathy. Ophthalmology *116*, 304–310.

Korte, G.E., Reppucci, V., and Henkind, P. (1984). RPE destruction causes choriocapillary atrophy. Invest. Ophthalmol. Vis. Sci. *25*, 1135–1145.

Lanza, R., Gearhart, J., Hogan, B., Melton, D., Pedersen, R., Thomas, E.D., Thomson, J., and Wilmut, I. (2009). Essentials of Stem Cell Biology, Second Edition (San Diego, CA: Academic Press/Elsevier).

Lee, K.Y., Vithana, E.N., Mathur, R., Yong, V.H., Yeo, I.Y., Thalamuthu, A., Lee, M.W., Koh, A.H., Lim, M.C., How, A.C., et al. (2008). Association analysis of CFH, C2, BF, and HTRA1 gene polymorphisms in Chinese patients with polypoidal choroidal vasculopathy. Invest. Ophthalmol. Vis. Sci. *49*, 2613–2619.

Leonard, D.S., Zhang, X.G., Panozzo, G., Sugino, I.K., and Zarbin, M.A. (1997). Clinicopathologic correlation of localized retinal pigment epithelium debridement. Invest. Ophthalmol. Vis. Sci. *38*, 1094–1109.

Lu, B., Malcuit, C., Wang, S., Girman, S., Francis, P., Lemieux, L., Lanza, R., and Lund, R. (2009). Long-term safety and function of RPE from human embryonic stem cells in preclinical models of macular degeneration. Stem Cells *27*, 2126–2135.

Lund, R.D., Wang, S., Klimanskaya, I., Holmes, T., Ramos-Kelsey, R., Lu, B., Girman, S., Bischoff, N., Sauvé, Y., and Lanza, R. (2006). Human embryonic stem cell-derived cells rescue visual function in dystrophic RCS rats. Cloning Stem Cells *8*, 189–199.

Maguire, A.M., Simonelli, F., Pierce, E.A., Pugh, E.N., Jr., Mingozzi, F., Bennicelli, J., Banfi, S., Marshall, K.A., Testa, F., Surace, E.M., et al. (2008). Safety and efficacy of gene transfer for Leber's congenital amaurosis. N. Engl. J. Med. *358*, 2240–2248.

Mori, K., Gehlbach, P.L., Kabasawa, S., Kawasaki, I., Oosaki, M., Iizuka, H., Katayama, S., Awata, T., and Yoneya, S. (2007). Coding and noncoding variants in the CFH gene and cigarette smoking influence the risk of age-related macular degeneration in a Japanese population. Invest. Ophthalmol. Vis. Sci. *48*, 5315–5319.

Ng, T.K., Chen, L.J., Liu, D.T., Tam, P.O., Chan, W.M., Liu, K., Hu, Y.J., Chong, K.K., Lau, C.S., Chiang, S.W., et al. (2008). Multiple gene polymorphisms in the complement factor h gene are associated with exudative age-related macular degeneration in chinese. Invest. Ophthalmol. Vis. Sci. *49*, 3312–3317.

Pei, X.T., Li, X.X., Bao, Y.Z., Yu, W.Z., Yan, Z., Qi, H.J., Qian, T., and Xiao, H.X. (2009). Association of c3 gene polymorphisms with neovascular age-related macular degeneration in a chinese population. Curr. Eye Res. *34*, 615–622.

Schatz, H., and McDonald, H.R. (1989). Atrophic macular degeneration. Rate of spread of geographic atrophy and visual loss. Ophthalmology *96*, 1541–1551.

Schwartz, S.D., Hubschman, J.P., Heilwell, G., Franco-Cardenas, V., Pan, C.K., Ostrick, R.M., Mickunas, E., Gay, R., Klimanskaya, I., and Lanza, R. (2012). Embryonic stem cell trials for macular degeneration: a preliminary report. Lancet *379*, 713–720.

Schwartz, S.D., Regillo, C.D., Lam, B.L., Eliott, D., Rosenfeld, P.J., Gregori, N.Z., Hubschman, J.P., Davis, J.L., Heilwell, G., Spirn, M., et al. (2015). Human embryonic stem cell-derived retinal pigment epithelium in patients with age-related macular degeneration and Stargardt's macular dystrophy: follow-up of two open-label phase 1/2 studies. Lancet *385*, 509–516.

Strauss, O. (2005). The retinal pigment epithelium in visual function. Physiol. Rev. *85*, 845–881.

Tsang, W.K., Tong, K.L., Yeung, S., Lee, W., and Chan, H.W. (2000). Efficacy and safety of mycophenolate mofetil in different dosages in Asian renal allograft recipients. Transplant. Proc. *32*, 1755–1756.

van Meurs, J.C., ter Averst, E., Hofland, L.J., van Hagen, P.M., Mooy, C.M., Baarsma, G.S., Kuijpers, R.W., Boks, T., and Stalmans, P. (2004). Autologous peripheral retinal pigment epithelium translocation in patients with subfoveal neovascular membranes. Br. J. Ophthalmol. *88*, 110–113.

Wimmers, S., Karl, M.O., and Strauss, O. (2007). Ion channels in the RPE. Prog. Retin. Eye Res. *26*, 263–301.

Wickham, L., and Gregor, Z. (2013). Epiretinal membranes. In Retina, Fifth Edition, S.J. Ryan, ed. (Elsevier), pp. 1954–1961.

Stem Cell Reports, Volume *4*

Supplemental Information

Treatment of Macular Degeneration Using Embryonic Stem Cell-Derived Retinal Pigment Epithelium: Preliminary Results in Asian Patients

Won Kyung Song, Kyung-Mi Park, Hyun-Ju Kim, Jae Ho Lee, Jinjung Choi, So Young Chong, Sung Han Shim, Lucian V. Del Priore, and Robert Lanza

Figure S1.

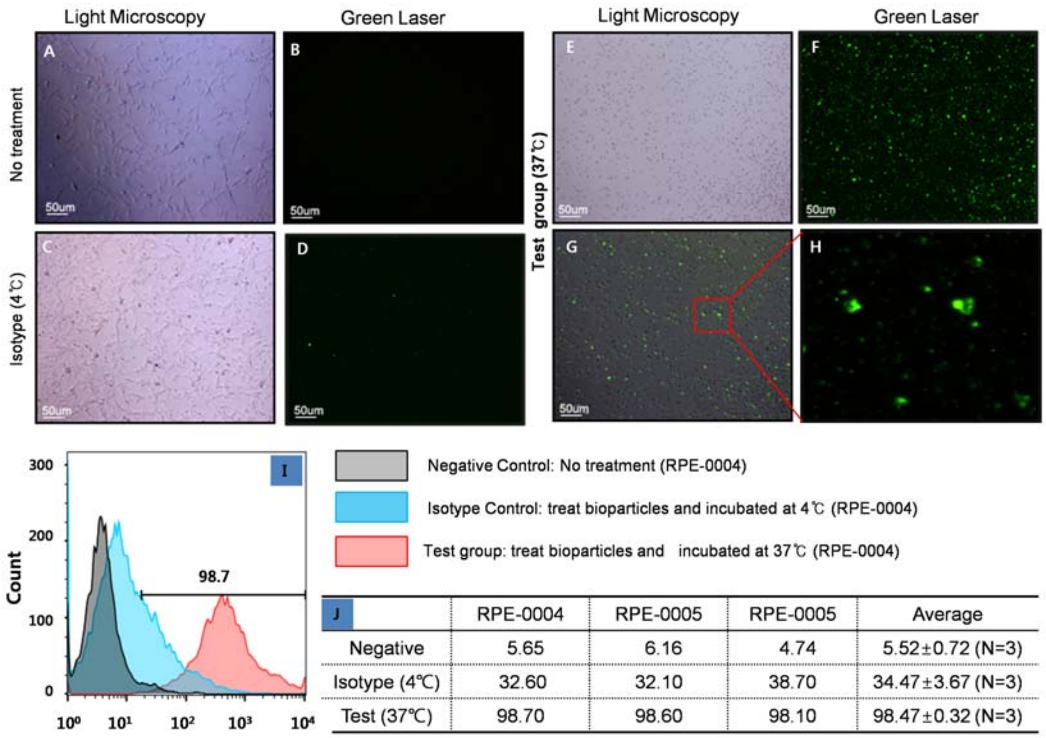

J	RPE-0004	RPE-0005	RPE-0005	Average
Negative	5.65	6.16	4.74	5.52±0.72 (N=3)
Isotype (4℃)	32.60	32.10	38.70	34.47±3.67 (N=3)
Test (37℃)	98.70	98.60	98.10	98.47±0.32 (N=3)

Figure S1. Quantification of Phagocytosis Assay Using FACS Analysis, Related to Results section. Frozen vials of final product were thawed and divided into three experiment groups: No treatment (A, B); treated with fluorescent *E.coli* bioparticles and incubated at 4℃ (C, D); and treated with fluorescent *E.coli* bioparticles and incubated at 37℃ (E-H). Phagocytized resuts of the hRPE cells were shown in the image of light microscopy (E), green laser (F) and merged in (G). Insert reveal magnified area for internalization of bioparticles clearly shown in (H). FACS analysis results of lot# RPE-0004 shown in (I) as an representative and the quantification of phagocytosis assay for the 3 lots of hRPE products shown in table (J). Scale bars: 50um.

Table S1. Schedule of Assessments for AMD Study, Related to Experimental Procedures Section.

Study Day / Assessment	Screening	Baseline	Day of Transplant	Post-transplant Assessments													
	-30 to -1D	-7 to -1D	D 0	Day 1	Day 3	Day 7±1D	Week 2±3D	Week 3±3D	Week 4±3D	Week 6±5D	Week 8±5D	Week 13±5D	Week 15±5D	Week 17±5D	Week 26±7D	Week 39±7D	Week 52±7D
Sign informed consent	X																
Assess inclusion/exclusion	X	X															
Blood collection for xenogeneic transplantation archiving		X															
Medical history	X	X	X	X	X	X	X	X	X	X	X	X	X	X	X	X	X
Review of adverse events		X	X	X	X	X	X	X	X	X	X	X	X	X	X	X	X
Concomitant medications	X	X	X	X	X	X	X	X	X	X	X	X	X	X	X	X	X
Physical examination, vital signs	X			X	X	X	X	X	X	X	X	X	X	X	X	X	X
Serum pregnancy test	X	X															X
Clinical laboratory tests	X			X	X	X	X	X	X	X	X	X	X	X	X	X	X
Cancer screening	X																
Chest x-ray	X																
ECG	X			X							X	X			X	X	X
Visual acuity	X	X		X	X	X	X	X	X	X	X	X	X	X	X	X	X
Refraction	X	X		X	X	X	X	X	X	X	X	X	X	X	X	X	X
Tonometry	X	X		X	X	X	X	X	X	X	X	X	X	X	X	X	X
NEI visual function questionnaire VFQ-25	X	X						X	X	X	X	X		X			X
Full dilated slit lamp evaluation	X	X		X	X	X	X	X	X	X	X	X	X	X	X	X	X
Indirect ophthalmoscope	X	X		X	X	X	X	X	X	X	X	X	X	X	X	X	X
Visual field testing	X	X					X	X	X	X	X	X		X	X	X	X
Spectral Domain OCT		X		X		X	X	X	X	X	X	X	X	X	X	X	X
Autofluorescence photography		X							X			X			X		X
Fundus photography		X		X				X	X	X	X	X	X	X	X	X	X
Fluorescein angiography		X						X	X		X	X		X	X	X	X
Electroretinogram		X										X			X		X
Reading speed		X							X			X			X		X
Axial Length Measurement	X																
Tacrolimus administration		X	X	X	X	X	X	X	X								
Tacrolimus blood		X	X	X	X	X	X	X	X								
Mycophenolate mofetil administration		X	X	X	X	X	X	X	X	X	X	(X)	(X)	(X)	(X)	(X)	(X)
Hospitalization		X	X	X													

Table S2. Schedule of Assessments for the SMD Study, Related to Experimental Procedures Section.

Study Day / Assessment	Screening	Baseline	Day of Transplant	Post-transplant Assessments											
	-30 to -1D	-7 to -1D	D 0	Day 1	Day 3	Day 7±1D	Week 2±3D	Week 3±3D	Week 4±3D	Week 6±5D	Week 8±5D	Week 13±5D	Week 26±7D	Week 39±7D	Week 52±7D
Sign informed consent	X														
Assess inclusion/exclusion	X	X													
Blood collection for xenogeneic transplantation archiving		X													
Medical history	X	X	X	X	X	X	X	X	X	X	X	X	X	X	X
Review of adverse events		X	X	X	X	X	X	X	X	X	X	X	X	X	X
Concomitant medications	X	X	X	X	X	X	X	X	X	X	X	X	X	X	X
Physical examination, vital signs	X			X	X	X	X	X	X	X	X	X	X	X	X
Serum pregnancy test	X	X													X
Clinical laboratory tests	X			X	X	X	X	X	X	X	X	X	X	X	X
Cancer screening	X														
Chest x-ray	X														
ECG	X			X							X	X		X	X
Visual acuity	X	X		X	X	X	X	X	X	X	X	X	X	X	X
Refraction	X	X		X	X	X	X	X	X	X	X	X	X	X	X
Tonometry	X	X		X	X	X	X	X	X	X	X	X	X	X	X
NEI visual function questionnaire VFQ-25	X	X						X	X	X	X	X			X
Full dilated slit lamp evaluation	X	X		X	X	X	X	X	X	X	X	X	X	X	X
Indirect ophthalmoscope	X	X		X	X	X	X	X	X	X	X	X	X	X	X
Visual field testing	X	X					X	X	X	X	X	X		X	X
Spectral domain OCT		X		X		X	X	X	X	X	X	X	X	X	X
Autofluorescence photography		X							X			X			X
Fundus photography		X		X				X	X	X	X	X	X	X	X
Fluorescein angiography		X						X	X		X	X		X	X
Electroretinogram		X										X			X
Tacrolimus administration		X	X	X	X	X	X	X	X						
Tacrolimus blood		X	X	X	X	X	X	X	X						
Mycophenolate mofetil administration		X	X	X	X	X	X	X	X	X	X	(X)	(X)	(X)	
Hospitalization		X	X	X											

Supplemental Experimental Procedures

Phagocytosis Assay, Related to Results section.

Fluorescent *E.coli* Bioparticles (Invistrogen Cat #V6694) suspension was prepared prior to

phagocytosis assay according to the manufacture's instruction. Differentiated hRPE cells during the culture in a 6-well plate were incubated with 600μl/well fluorescent labeled bioparticles overnight at 37℃. After washing cells twice with 2ml of phosphate-buffered saline (PBS pH7.4), 2ml of cell culture medium was added to each well and incubated at 37℃ for 2 days. For the final product, frozen vials were thawed, washed twice with culture medium and cells were divided into three 15ml Falcon tubes. 200ul cell culture medium was added for one tube and 200ul fluorescent *E.coli* bioparticles were added for the other two tubes. Except isotype negative control tube, the other tubes were incubated at 37℃ for overnight. As for an isotype negative control tube, which was treated with 200μl fluorescent labeled bioparticles, was incubated at 4℃. After removing the biopartlcles solutions, cells were washed twice with 10ml cell culture medium, seeded 5x10^{5} cells/well of 6-well plate and incubated at 37℃ for 2 days. For fluorescence observation, the cells were washed twice with 2ml of phosphate-buffered saline (PBS pH 7.4) and washed once with 1ml purified water. The cells then were fixed with 400μl of 4% paraformaldehyde solution for 5-10 minutes. The cells were examined under the fluorescence microscopy (Nikon ECLIPSE Ti-U, S/N: 633088) after removing fixative solution and were washed twice with 100μl phosphate-buffered saline (PBS pH 7.4). Photo images were taken by using NIS-Elements BR (version:3.2.3) software. For quantification analysis of the phagocytosis, the rest of the cells in the 6-well plate were trypsinized with 0.25% Trypsin-EDTA and underwent FACS analysis using Guaca easyCyteTM Dual Laser System and Guva Software (version 2.2.3).

FACS analysis, Related to Results section.

For OCT-4 staining, 1 x 10^{5} cells of each sample were washed with 10% FBS/DMEM:F12 and resuspended in 1ml 4% paraformaldehyde/PBS and incubated for 10min at room temperature. The cells were washed twice with 10% FBS/DMEM:F12 and resuspended in 0.1% Triton X-100/PBS and incubated for 15 minutes on ice. After washing twice with 10% FBS/DMEM:F12, the cells were resuspended in 95μl phosphate-buffered saline (PBS; pH 7.4) and incubated with 0.1 μg of the primary antibody (OCT-3/4-Alex 488, BD Biosciences cat# 560217) against OCT-3/4 for 30~60 minutes at 4°C in dark. For TRA-1-60 staining, the cells were washed twice with 1 ml PBS supplemented with 1% heat inactivated FBS. 1 x 10^{5} cells of each sample were resuspended in 95 μl phosphate-buffered saline (PBS; pH 7.4) and incubated with 0.1 μg of the primary antibody (TRA-1-

60-PE, BD Biosciences cat #560193) against TRA-1-60 for 30~60 minutes at 4°C in dark. The negative controls used for FACS gating were Mouse IgG1K Isotype Control FITC (e-Bioscience cat#12-4714) and Mouse IgG1K Isotype Control PE (e-Bioscience cat#12-4714). Each sample was washed twice with 1 ml PBS supplemented with 1% heat inactivated FBS. Finally, the cells were resuspended in 400 µl PBS and subsequently analyzed by flow cytometry using Guaca easyCyteTM Dual Laser System and Guava soft software(version 2.2.3). The sample flow rate during analysis did not exceed 300-400 cells per second.

Immunocytochemistry, Related to Results section.

Cells were fixed with 4 % paraformaldehyde for 5-10 minutes and permeabilized with 0.2 % Triton x-10 in PBS for 10 minutes. Unspecific binding was blocked in PBS supplemented with 10 % goat serum for 1-2 hours, and incubated with primary antibodies for 1 hour at room temperature or overnight at 4°C in blocking buffer. After washing twice with phosphate-buffered saline (PBS; pH 7.4), the cells were incubated with fluorescent secondary antibodies Alexa Fluor® 488 conjugate or Alexa Fluor® 594 conjugate (Invitrogen cat# A11008, cat# A11005) for 1 hour at room temperature. Nuclei were stained with 4'-6-Diamidino-2-phenylindole (DAPI, Sigma cat# D9564) for 5 min at room temperature. The cells were then examined under the fluorescence microscopy (Nikon ECLPSE Ti-U, S/N:633088). Photo images were taken by using NIS-Elements BR (version:3.2.3) software. Primary antibodies for the staining as follows: ZO-1 (Invitrogen cat# 40-2300), PAX-6 (Millipore cat# AB2237), MITF (Thermo cat# MS-772-p), Bestrophin (Novus Biologicals cat# NB300-164), OCT-3/4 (SantaCruz cat# SC5279), NANOG (CellSignaling #3580S).

Real-time PCR, Related to Results section.

The RNeasy RNA isolation kit form Qiagen was used to extract total cellular RNA, and cNDA was synthesized from the RNA with the cDNA synthesis kit from Qiagen. Quantitative real-time PCR was performed using the Roche LihgtCycler 480/96 Quantitative Real-Time PCR Instrument and TaqMan® Gene Expression Maste Mix, following manufacturer's cycle conditions. *NANOG, OCT-4* and *SOX2* for hES markers and *RPE-65, PAX-6, MITF* and *BESTROPHIN* for hRPE markers were

analyzed. Primers for hES and hRPE markers were generated by Ocata therapeutics Inc.' as custom-made inventoried from Applied Biosystems (*NANOG*: cat# Hs02387400, *OCT-4*: cat# Hs03005111, *SOX-2*: cat# Hs01053049, *RPE-65*: cat#Hs01071462, *PAX-6*: cat#Hs00240871, *MITF*: cat#Hs01117294, *Bestrophin*: cat#Hs00188249). As an endogenous control, human *beta ACTIN* was used (Applied Biosystems: cat# 4333762T).

Safety analysis, Related to Results section.

For adventitious viruses test, we used the methods of adsorption of hemagglutininis virus by red blood cell. Endotoxin test was measured by EndoScan-V (version 4.0 and higher) with the Endosafe®-MSC kinetic Reader (Charles River Laboratories, Model no. MCS Reader, S/N: 0314). Releasing specification for final product is < 0.5 EU/ml and the result for clinical product is normally < 0.32 EU/ml. MAP test for murine viruses was conducted by Charles River Laboratories (251 Ballardvale Street Wilmington, MA01887 USA) for the three different lots.

Viability test after final formulation, Related to Results section.

Live cells were counted in a hemocytometer using 0.4% trypan blue dye for measure viability of cells. After final washing in BSS plus solution, the cells were suspended in about 200μl and count the cells to make 2,000 cells/μl. And then, the cells were manipulated to loading density 444 cells/μl targeting for the clinical does 50,000 cells/150μl. Final formulated cells were stored at 2-8□ until clinical use up to 4 hours.

Inclusion/Exclusion Criteria for AMD Study, Related to Experimental Procedures Section.

Inclusion Criteria:

1. Adult male or female older than 55 years of age.
2. Patient should be in sufficiently good health to reasonably expect survival for at least 4 years.
3. Clinical findings consistent with advanced dry age-related macular degeneration (AMD) with evidence of one or more areas of >250 microns of geographic atrophy (as defined in the Age-related Eye Disease Study [AREDS] study) involving the central fovea.

4. Geographic atrophy (GA) defined as attenuation or loss of RPE as observed by slit-lamp biomicroscopy, optical coherence tomography (OCT), and fluorescein angiography (FA).
5. The visual acuity (Best Corrected Visual Acuity [BCVA]) of the eye to receive the transplant will be no better than 20/400.
6. The visual acuity (BCVA) of the eye that is NOT to receive the transplant will be no worse than 20/400.
7. Electrophysiological findings consistent with advanced dry AMD.
8. Medically suitable to undergo vitrectomy and subretinal injection.
9. Medically suitable for general anesthesia.
10. If female and of childbearing potential, willing to use medically effective forms of birth control during the study.

    *Medically effective method of contraception: condoms, continuous use oral contraceptives for more than 3 months, injection or insertion of contraceptives, and insertion of an intrauterine device (IUD).
11. If male, willing to use medically effective contraception during the study.
12. Willing to defer all future blood, blood components or tissue donations.
13. Able to understand and willing to sign the informed consent form.

Exclusion Criteria:

1. Presence of active or inactive choroidal neovascularization(CNV).
2. Presence or history of retinal dystrophy, retinitis pigmentosa, chorioretinitis, central serous chorioretinopathy, diabetic retinopathy or other retinal vascular or degenerative disease other than AMD.
3. History of optic neuropathy
4. Presence of macular atrophy due to causes other than AMD.
5. Presence of glaucomatous optic neuropathy in the study eye, uncontrolled intraocular pressure (IOP), or the use of two or more agents to control IOP (acetazolamide, beta blocker, alpha-1-agonist, prostaglandins, carbonic anhydrase inhibitors).
6. Cataract of sufficient severity likely to necessitate surgical extraction within 1 year.
7. History of retinal detachment repair in the study eye.

8. Axial myopia of greater than -8 diopters.
9. Axial length greater than 28 mm.
10. Any other sight-threatening ocular disease.
11. Any history of retinal vascular disease (compromised blood-retinal barrier).
12. History of glaucoma.
13. Uveitis or other intraocular inflammatory disease.
14. Significant lens opacities or other media opacity.
15. Ocular lens removal within previous 3 months.
16. Ocular surgery in the study eye in the previous 3 months
17. History of malignancy.
18. Medically not suitable for transplantation of an embryonic stem cell line: Any laboratory value that falls slightly outside of the normal range will be reviewed by the medical monitor and investigators to determine its clinical significance.
    1) History of drug abuse
    2) Positive human immunodeficiency virus (HIV), hepatitis B (HBV), and hepatitis C (HCV) serologies.
    3) Any immunodeficiency.
    4) Positive cancer screening test
        - Medical history & physical examination
        - Chest X-ray
        - Complete blood count (CBC)
        - Routine urinalysis (U/A)
        - Thyroid exam (T3, T4, TSH, thyroid ultrasonography)
        - If male, testicular examination (scrotal ultrasonography, LDH, beta-HCG)
        - If male, digital rectal examination (DRE) and prostate specific antigen (PSA) testing
        - Stomach cancer examination (upper gastrointestinal series or esophagogastroduodenoscopy
        - Liver cancer examination (abdomen ultrasonography+ α-fetoprotein)

- Colorectal cancer examination (occult blood in feces + colonoscopy or colon study)
- Breast cancer examination (mammography, breast ultrasonography, breast physical examination)
- Uterine cervical cancer examination (sonohysterography, Pap Smear, pelvis examination)

5) History of diabetes mellitus.
   - Fasting plasma glucose ≥126 mg/dL
   - Typical symptoms of diabetes and random plasma glucose ≥200 mg/dL
   - HbA1c ≥7%
6) Alanine transaminase/aspartate aminotransferase (ALT/AST) >1.5 times the upper limit of normal or any known liver disease
7) Renal insufficiency, as defined by creatinine level >1.3 mg/dL.
8) A hemoglobin concentration < 10 gm/dL, a platelet count < 100k/mm^3 or an absolute neutrophil count < 1000/mm^3 at study entry.

19. History of myocardial infarction or cerebral vascular disease in the previous 12 months.
20. History of cognitive impairments or dementia that may impact the patient’s ability to participate in the informed consent process and to appropriately complete evaluations.
21. Any current immunosuppressive therapy other than intermittent(if continuously, < 14 days)or low-dose corticosteroids (prednisolone ≤ 10 mg/day, dexamethasone ≤ 10 mg/day)
22. Current participation in any other clinical trial.
23. Participation within the previous 6 months in any clinical trial of a drug by ocular or systemic administration.
24. If female, pregnancy or lactation.
25. Any other medical condition, which, in the investigator's judgment, will interfere with the patient's ability to comply with the protocol, compromises patient safety, or interferes with the interpretation of the study results.

Inclusion/Exclusion Criteria for the SMD Study, Related to Experimental Procedures Section.

Inclusion Criteria:

1. Adult male or female over 20 years of age.
2. Clinical diagnosis of advanced SMD.
3. Visual acuity (BCVA) of the eye to receive the transplant will be no better than 20/400.
4. Visual acuity (BCVA) of the eye that is NOT to receive the transplant no better than 20/400.
5. Peripheral visual field constriction documented on standard visual field testing.
6. Electrophysiological findings consistent with SMD.
7. Medically suitable to undergo vitrectomy and subretinal injection.
8. Medically suitable for general anesthesia.
9. If female and of childbearing potential, willing to use medically effective forms of birth control during the study.

   *Medically effective methods of contraception : condoms, continuous use oral contraceptives for more than 3 months, injection or insertion of contraceptives, insertion of an intrauterine device (IUD)

10. If male, willing to use medically effective contraception during the study.
11. Willing to defer all future blood, blood component or tissue donations.
12. Able to understand and willing to sign the informed consent form.

Exclusion Criteria:

1. History of malignancy.
2. History of myocardial infarction or cerebrovascular disease in the previous 12 months.
3. Medically not suitable for transplantation of an embryonic stem cell line: Any laboratory value that falls slightly outside of the normal range will be reviewed by the medical monitor and investigators to determine its clinical significance.
   1) History of drug abuse
   2) Positive human immunodeficiency virus (HIV), hepatitis B (HBV), and hepatitis C (HCV) serologies.
   3) Any immunodeficiency.
   4) Positive cancer screening test

- Medical history & physical examination
- Chest X-ray
- Complete blood count (CBC)
- Routine U/A
- Thyroid examination (T3, T4, TSH, thyroid ultrasonography)
- If male, testicular examination
  (scrotal ultrasonography , LDH, beta-HCG)
- If male older than 40 years, digital rectal examination (DRE) and prostate-specific antigen (PSA) testing
- Stomach cancer examination (upper gastrointestinal series or esophagogastroduodenoscopy)
- Liver cancer examination (abdomen ultrasonography+ α-fetoprotein testing)
- Colorectal cancer examination (occult blood in feces + colonoscopy or colon study)
- Breast cancer examination (mammography, breast ultrasonography, breast physical examination)
- Uterine cervical cancer examination (sonohysterography, Pap Smear, pelvis examination)

5) History of diabetes mellitus
   -Fasting plasma glucose ≥126 mg/dL
   - Typical symptoms of diabetes and random plasma glucose ≥200 mg/dL
   -HbA1c ≥7%
6) Alanine transaminase/aspartate aminotransferase (ALT/AST) >1.5 times the upper limit of normal or any known liver disease
7) Renal insufficiency, as defined by a creatinine level >1.3 mg/dL.
8) A hemoglobin concentration <10 gm/dL, a platelet count <100 k/mm3 or an absolute neutrophil count <1000/mm3 at study entry.

4. Any current immunosuppressive therapy other than intermittent (if continuously <14 days) or low dose corticosteroids (prednisolone ≤ 10 mg/day, dexamethasone ≤ 10mg/day)
5. Current participation in any other clinical trial.
6. Participation within the previous 6 months in any clinical trial of a drug by ocular or systemic administration.

7. Any other sight-threatening ocular disease
8. Any chronic ocular medications.
9. Any history of retinal vascular disease (compromised blood-retinal barrier.)
10. Glaucoma.
11. Uveitis or other intraocular inflammatory disease.
12. Significant lens opacities or other media opacity
13. Ocular lens removal within the previous 3 months.
14. If female, pregnant or lactating
15. Any other medical condition that, in the investigator's judgment, will interfere with the patient's ability to comply with the protocol, compromises patient safety, or interferes with the interpretation of the study results.
